# Sub‐Second Ratiometric Fluorescence Imaging of Histamine Dynamics in Living Cells via a Supramolecular Probe

**DOI:** 10.1002/advs.78254

**Published:** 2026-10-14

**Authors:** Chenming Chan, Yujie Han, Yu‐Fei Song, Chunlei Jiang, Qi‐Wei Zhang

**Affiliations:** ^1^ Department of Chemistry School of Chemistry and Molecular Engineering East China Normal University Shanghai P. R. China; ^2^ Institute of Magnetic Resonance and Molecular Imaging in Medicine East China Normal University Shanghai P. R. China

**Keywords:** fluorescence imaging, histamine, ratiometric, sub‐second resolution, supramolecular probe

## Abstract

Histamine (HA) is a critical neurotransmitter, yet its intracellular dysregulation remains difficult to monitor due to the slow kinetics and environmental sensitivity of traditional fluorescent probes. Herein, we report a supramolecular ratiometric probe engineered via the host–guest self‐assembly of cationic fluorophores with cucurbit[7]uril (CB[7]). By precisely modulating the cationic locus within the guests, we achieved a 2:1 (host:guest) stoichiometric complex (**CB[7]_2_⊃GA‐1**) that displays a distinct 60 nm bathochromic shift. Functioning via a competitive displacement mechanism, this probe demonstrates exceptional selectivity and an ultrafast response time of 0.96 s (*k*_obs_ = 3.56 s^−^^1^). We successfully utilized this system to visualize real‐time HA fluctuations in living mast cells with sub‐second resolution. Notably, our imaging revealed a quantitative correlation between nigericin‐induced cytoplasmic HA elevation and mast cell rupture. These findings validate the probe's efficacy in tracking cellular inflammatory responses and highlight its potential as an early‐warning indicator for drug‐induced cytotoxicity. Consequently, this work offers a robust tool for dissecting HA‐related pathophysiology and establishes a generalizable design paradigm for the rapid, ratiometric sensing of biologically relevant analytes.

## Graphical abstract

A tailored tripartite supramolecular ratiometric probe, engineered by modulating the fluorogenic guest's cationic locus, enables ultrafast histamine monitoring in living cells. Utilizing a competitive displacement mechanism, this system achieves a sub‐second response time with high selectivity. Real‐time imaging successfully visualized histamine fluctuations in mast cells, establishing a quantitative correlation between cytoplasmic histamine elevation and drug‐induced degranulation followed by cell rupture.

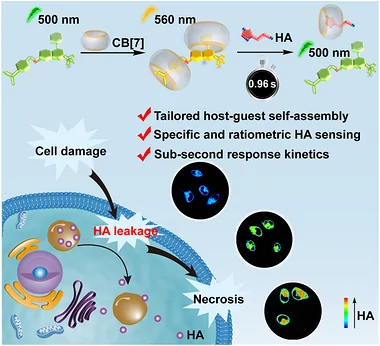

## 1 Introduction

Histamine (HA), a biogenic amine synthesized from L‐histidine via decarboxylation by histidine decarboxylase, serves as a pivotal signaling molecule in a myriad of physiological activities, including sleep‐wake cycles, learning and memory, emotional stress, dietary intake, and thermoregulation. [1, 2, 3]. Mast cells are the primary producers of HA, which is synthesized in the Golgi apparatus, stored in secretory granules, and subsequently transported and secreted into the extracellular space to participate in immune regulation and neuroinflammatory responses [4]. Altered HA dynamics within brain mast cells are closely linked to variations in brain parenchyma, potentially inducing neurodegenerative pathologies such as Alzheimer's disease and epilepsy [5, 6, 7]. While HA remains stable within secretory granules under physiological conditions, pathological states—such as mast cell necrosis or the disruption of granule homeostasis—trigger the rapid release of granular contents into the cytoplasm [8, 9, 10]. This release serves as a critical marker of impaired cell function and the progression of necrosis. Consequently, there is an urgent need to develop molecular probes capable of rapid, quantitative visualization of HA dynamics in living cells.

Fluorescent probe‐based imaging represents a premier approach for monitoring bioactive molecules in situ [11, 12, 13, 14, 15, 16, 17, 18, 19, 20, 21]. Although genetically encoded indicators have been engineered to detect HA release in live systems, their utility is often constrained by challenges in organelle‐specific targeting, difficulties in quantitative detection, and potential risks associated with genetic manipulation [22]. In this context, small‐molecule probes offer distinct advantages due to their superior biocompatibility [23, 24, 25, 26, 27, 28, 29, 30]. However, currently reported small‐molecule fluorescent probes predominantly rely on single‐wavelength channels (Table S1), rendering them susceptible to artifacts arising from uneven probe distribution and excitation light fluctuations in complex biological systems (such as live cells, tissues, or in vivo imaging) [31, 32]. Furthermore, existing HA fluorescent probes are mostly based on covalent reactions that generally suffer from slow kinetics, making it difficult to capture the rapid, transient variations of HA levels characteristic of physiological signaling [33].

Herein, we report the design and synthesis of a series of fluorescent guest molecules (**GA‐1** to **GA‐3**) that self‐assemble with the macrocycle cucurbit[7]uril (CB[7]) (Figure 1). By strategically modulating the position of the cationic locus on the guest molecules (Figure 1B), we achieved precise control over the stoichiometry and structure of the resulting supramolecular complexes in aqueous media. Specifically, **GA‐1**, which features a central positive charge, spatially separates two aromatic anchoring sites. This arrangement facilitates the formation of a 1:2 complex (**CB[7]_2_⊃GA‐1**) via entropy‐driven self‐assembly. In contrast, for guests **GA‐2** and **GA‐3**, the terminal positive charges direct a single CB[7] macrocycle to bind at the molecular center. The resulting steric hindrance precludes the binding of a second macrocycle, leading to a 1:1 stoichiometry. These distinct assembly modes elicit unique spectral responses, with only **GA‐1** exhibiting a red‐shifted intramolecular charge transfer (ICT) emission upon forming the 1:2 complex. Due to this ratiometric response, the **CB[7]_2_⊃GA‐1** assembly was identified as the optimal candidate for sensing applications. On the other hand, owing to its higher binding affinity for CB[7] compared to **GA‐1**, HA competitively displaces the guest molecule from the macrocyclic cavity. This displacement restores the original fluorescence of free **GA‐1**, generating a robust ratiometric signal. Benefiting from the rapid kinetics of non‐covalent interactions, the probe demonstrates a sub‐second response time (0.96 s) toward HA. Combined with excellent selectivity, photostability, and low biotoxicity, the supramolecular probe **CB[7]_2_⊃GA‐1** was successfully employed to dynamically monitor HA variations associated with abnormal mast cell function, elucidating a direct quantitative correlation between mast cell degranulation, HA release, and cellular morphology during swelling or rupture (Figure 1A).

**FIGURE 1:**
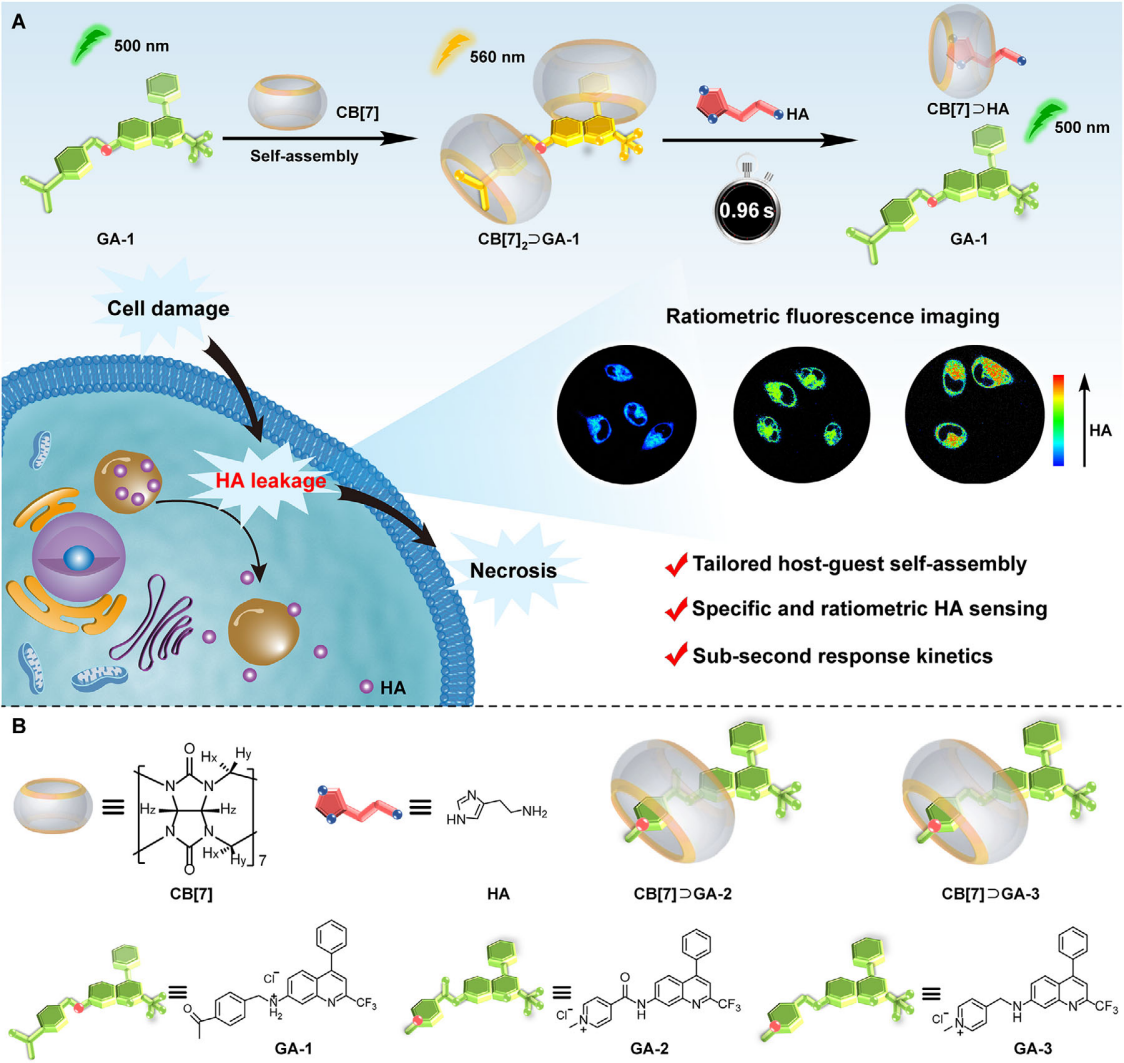
Design of the supramolecular probe for the visualization and quantification of histamine (HA) in mast cells. (A) Schematic illustration of the assembly and sensing mechanism of the probe **CB[7]_2_⊃GA‐1**, enabling specific and rapid imaging of HA. (B) Chemical structures of the host and guest molecules, along with the reference supramolecular complexes **CB[7]⊃GA‐2** and **CB[7]⊃GA‐3**.

## 2 Results and Discussion

### 2.1 Molecular Design and Structural Characterizations of the Supramolecular Complexes

Cucurbit[n]urils (CB[n]s) are a class of rigid macrocyclic molecules characterized by a hydrophobic cavity and electronegative carbonyl portals [34, 35, 36, 37, 38]. This unique structural architecture endows them with the ability to engage in hydrophobic inclusion with aromatic or aliphatic moieties, as well as charge–dipole interactions with cationic species. Herein, we designed a series of cationic fluorescent guest molecules, **GA‐1** to **GA‐3**, featuring orthogonal bis‐aromatic branches. The synthetic procedures and characterizations are detailed in Figures S1–S19. Driven by the quaternary ammonium cationic centers and the specific positioning of the positive charges, guest molecules **GA‐2** and **GA‐3** exhibited analogous self‐assembly modes with the CB[7] macrocycle. As shown in Figure S20, Job's plots indicated that both guest molecules formed 1:1 inclusion complexes with CB[7], denoted as **CB[**7**]⊃GA‐2** and **CB[**7**]⊃GA‐3**. The fine structure of the self‐assembled complex formed between the representative guest **GA‐2** and CB[7] was further investigated using ^1^H NMR and 2D ROESY NMR spectroscopy. As illustrated in Figure S21, compared to its free state, the pyridine protons of the guest molecule **GA‐2** shifted upfield upon complexation with CB[7], indicating enhanced shielding of this moiety. Furthermore, nuclear Overhauser effect (NOE) cross‐peaks between the pyridine protons H(1', 3') and CB[7] protons were observed in the 2D ROESY NMR spectrum, confirming their spatial proximity (Figure S22). This demonstrates that the self‐assembly between the macrocycle and the guest molecule occurs at the pyridinium branch, mediated by hydrophobic interactions between the pyridine and the CB[7] cavity, as well as electrostatic dipole interactions between the positive charge and the carbonyl portals of CB[7]. Concurrently, fluorescence spectroscopic results revealed that during titration with CB[7], **GA‐2** and **GA‐3** exhibited a moderate enhancement of their original emission peaks, with fluorescence quantum yields of 0.62% and 3.48%, respectively (Figure S23). This enhancement can be attributed to the suppression of non‐radiative transitions via macrocyclic inclusion.

In contrast, **GA‐1** formed a distinctly different assembly mode and structure with CB[7]. As depicted in Figure 2A, Job's plots revealed that **GA‐1** forms a 1:2 complex with CB[7] (denoted as **CB[7]_2_⊃GA‐1**). Fluorescence spectroscopic results further revealed that upon the gradual addition of CB[7], the fluorescence emission peak of the **GA‐1** solution underwent a distinct red shift from 500 to 560 nm (Figure 2B). A plot of the fluorescence intensity ratio at these two wavelengths against the concentration of CB[7] demonstrated that the ratiometric signal reached a plateau when the CB[7] concentration reached 2 equivalents relative to **GA‐1**, yielding a fluorescence quantum yield of 51.87%, further confirming the 1:2 binding stoichiometry (Figure 2C). Furthermore, electrospray ionization high‐resolution mass spectrometry (ESI‐HR‐MS) result provided clear evidence for the formation of a complex involving **GA‐1** and two equivalents of CB[7] (Figure 2D). To further characterize the specific spatial architecture of **CB[7]_2_⊃GA‐1**, ^1^H NMR spectroscopy was performed. As shown in Figure 2E, upon self‐assembly with CB[7], the protons H(1,2) and H(6–8) located on the dual branches of **GA‐1** exhibited significant upfield shifts, indicating that these phenyl protons experienced enhanced shielding effects. To achieve a more detailed structural characterization of the supramolecular complexes, 2D ROESY NMR spectroscopy was employed. As shown in Figure 2F, strong NOE cross‐peaks were observed between protons H(1', 2', 11', and 6') from the aromatic branches on both sides of **GA‐1** and protons H(x–z) of CB[7] [39, 40]. These correlations provide direct evidence of their spatial proximity within the complex, thereby confirming that two CB[7] molecules simultaneously encapsulated both aromatic moieties.

**FIGURE 2:**
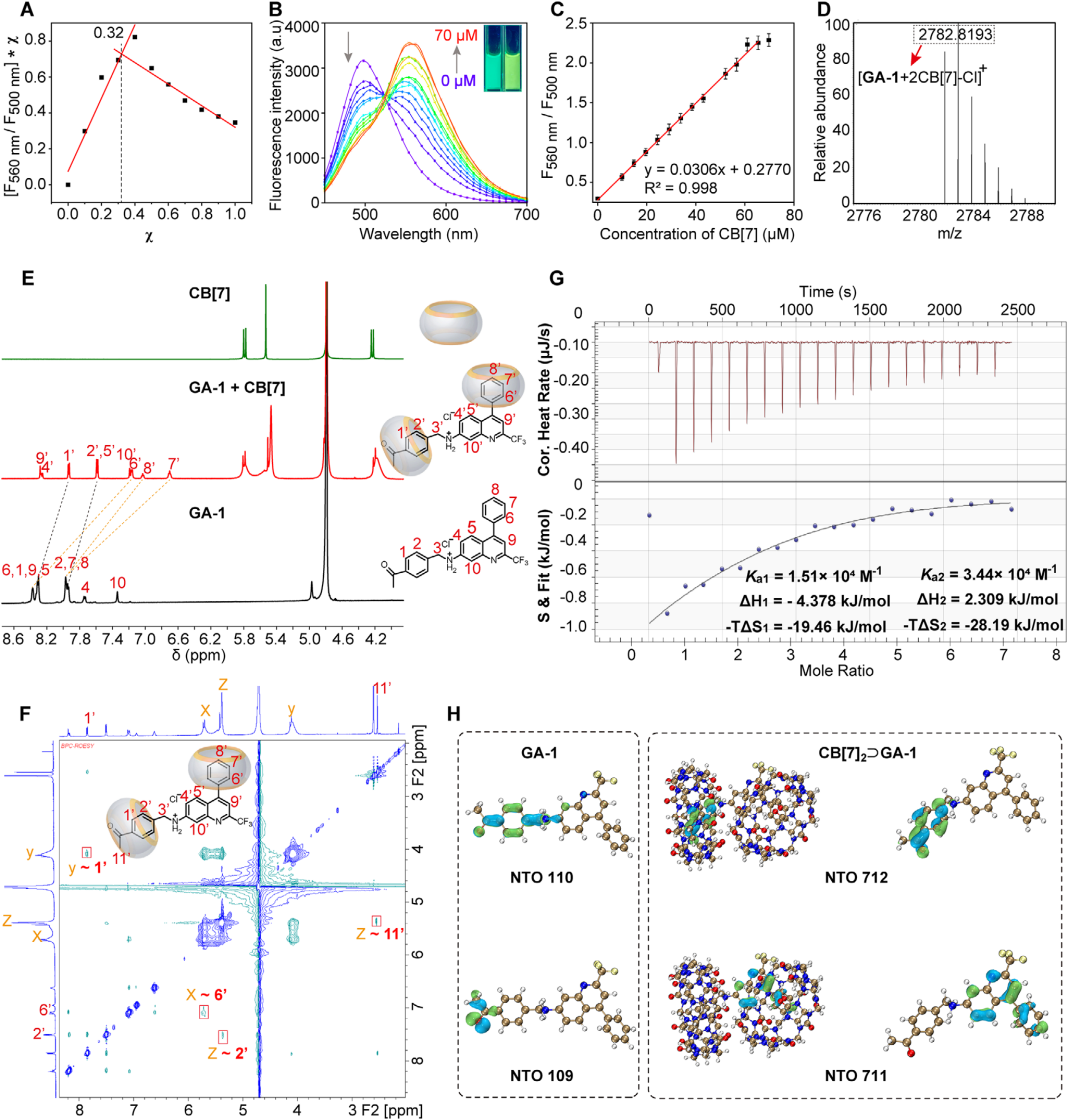
Multimodal structural analysis of the supramolecular complex **CB[7]_2_⊃GA‐1**. (A) Job's plot for the complexation between **GA‐1** and CB[7] (total concentration = 30 µm), derived from fluorescence data. χ = [**GA‐1**]/([**GA‐1**]+[CB[7]]). (B) Fluorescence emission spectra of **GA‐1** (30 µm) upon the addition of varying concentrations of CB[7] in aqueous solution, Ex = 440 nm. Inset: Photographs of the **GA‐1** solution under 365 nm UV illumination before (left) and after (right) the addition of CB[7]. (C) The linear relationship between the fluorescence intensity ratio (F_560 nm_/F_500 nm_) and the concentration of CB[7]. (D) ESI‐HR‐MS analysis of the supramolecular complex **CB[7]_2_⊃GA‐1**. (E) ^1^H NMR spectra of **GA‐1**, **CB[7]_2_⊃GA‐1**, and CB[7] in D_2_O at 25°C (0.5 mm). (F) Partial 2D ROESY NMR spectrum of **CB[7]_2_⊃GA‐1**. (G) ITC isotherm and thermodynamic fitting for the binding between **GA‐1** and CB[7] in water, [**GA‐1**] (cell) = 10 µm, CB[7] (syringe) = 100 µm. (H) Natural transition orbital (NTO) track plots of the guest **GA‐1** and the **CB[7]_2_⊃GA‐1** complex (CB[7] was omitted for clarity in the right‐side part).

The two distinct binding stoichiometries and complex structures described above correspond precisely to the structural characteristics of the guest molecules, specifically highlighting the decisive role of the cationic center's position in directing the self‐assembly mode. When the positive charge is located at the terminal end of the molecule (e.g., **GA‐2** and **GA‐3**), CB[7] preferentially encapsulates the pyridinium site on the primary branch. Since the positive charge is distal to the orthogonal aromatic branch in these guests, the driving force for potential CB[7] encapsulation on that side is weak. Furthermore, the confinement of the positive charge locks the CB[7] macrocycle at the central segment, positioning it in close proximity to the orthogonal benzene ring of the guest molecule. This creates steric hindrance that effectively prevents the inclusion of a second CB[7] molecule on the orthogonal branch (Figure 1). Conversely, when the positive charge is centrally located (e.g., **GA‐1**), electrostatic dipole interactions can simultaneously affect both orthogonal aromatic branches. This arrangement facilitates the self‐assembly of two separate CB[7] macrocycles without inducing significant steric hindrance (Figure 1). This inference is further supported by the thermodynamic parameters of the assembly process through isothermal titration calorimetry (ITC) studies. As shown in Figure 2G, the fitted thermodynamic titration curve reveals a dual‐inclusion process. Both steps exhibit high binding constants (*K*_a1_ = 1.51 × 10^4^ M^−^^1^, *K*_a2_ = 3.44 × 10^4^ M^−^^1^). This indicates that the initial assembly does not hinder subsequent macrocyclic encapsulation but rather exhibits a slight positive cooperative effect, driven primarily by a significant entropic contribution.

Following the demonstration of the 1:2 self‐assembly mode and thermodynamic parameters between **GA‐1** and CB[7], we further optimized the structure through density functional theory (DFT)‐based calculations and performed molecular orbital simulations. First, the vertical excitation energies corresponding to the transition from the ground state (S_0_) to the first excited state (S_1_) for both **GA‐1** and **CB[7]_2_⊃GA‐1** were calculated. For the guest molecule **GA‐1**, the calculated S_0_–S_1_ vertical excitation energy is 2.3124 eV, whereas this value decreases to 2.0462 eV for the **CB[7]_2_⊃GA‐1** complex. This reduction of 0.2662 eV clearly indicates that the excitation energy decreases upon host–guest complexation, which is consistent with the experimentally observed red shift in both absorption and emission spectra. Furthermore, to provide rigorous evidence for the proposed ICT character, we analyzed the electron and hole distributions via Natural Transition Orbital (NTO) analysis. As shown in Figure 2H, the hole and electron densities for the free guest **GA‐1** exhibit limited spatial separation and are primarily localized on the pyridine branch, indicative of short‐range ICT character. In contrast, the NTOs for the supramolecular complex **CB[7]_2_⊃GA‐1** display a significantly more pronounced spatial separation, with the hole and electron pairs localized on two perpendicular branches of the guest molecule. This distinct distribution confirms a long‐range ICT process, which rationalizes the further red‐shifted emission wavelength observed in the complex.

### 2.2 Competitive Displacement of the Supramolecular Complex CB[7]_2_⊃GA‐1 by HA

Given that the supramolecular complex **CB[7]_2_⊃GA‐1** exhibits dual‐wavelength ratiometric spectral responsiveness—superior to the simple turn‐on response of the other two guest complexes—it is particularly well‐suited for biosensing applications. Consequently, **CB[7]_2_⊃GA‐1** was selected as the optimal supramolecular probe to investigate its molecular recognition capabilities. Owing to its characteristic imidazole group and primary alkylamine moiety, HA possesses significant structural and electronic complementarity with CB[7]. Initially, ^1^H NMR spectroscopy was employed to evaluate the host‐guest interactions between HA and CB[7]. As shown in Figure S24, upon the addition of one equivalent of CB[7] to a deuterated aqueous solution of HA, the aromatic imidazole protons and adjacent methylene protons of HA exhibited significant upfield shifts. These observations indicate that these moieties are encapsulated within the CB[7] cavity, thereby experiencing distinct shielding effects. Furthermore, DFT‐based structural simulations suggest that the formation of the host‐guest self‐assembled complex is further facilitated and stabilized by two sets of hydrogen bonds established between the imidazole moiety and primary alkylamine of HA with the portal carbonyls of CB[7] (Figure 3A). Subsequently, ITC measurements were conducted to elucidate the thermodynamic parameters and driving forces governing the self‐assembly of HA and CB[7]. As illustrated in Figure 3B, the results confirmed that HA bound to CB[7] with a 1:1 stoichiometry (*n* = 1.005). This complexation process is characterized by a high binding constant of 6.58 × 10^5^ M^−1^ and is driven by a significant entropic contribution (−*T*Δ*S* = −31.28 kJ mol^−1^). Notably, this binding affinity is an order of magnitude greater than that of either branch of the guest molecule **GA‐1** with CB[7]. Thermodynamically, this indicates that the introduction of HA is sufficient to trigger the competitive displacement of the guest within the supramolecular probe.

**FIGURE 3:**
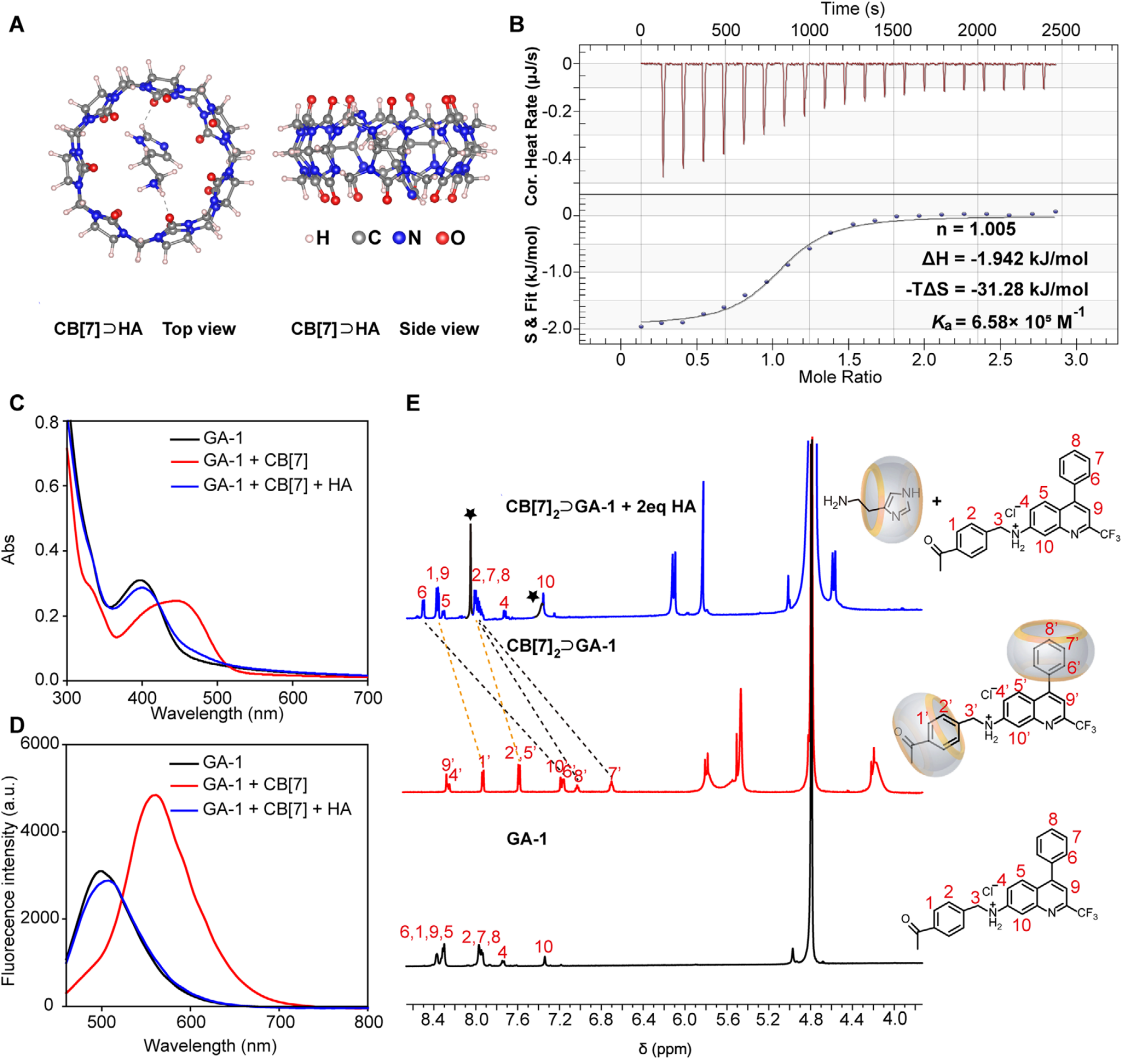
Characterization of host‐guest recognition mechanism of **CB[7]_2_⊃GA‐1** with HA. (A) Top and side views of the DFT‐optimized structure of the **CB[**7**]⊃HA** complex. (B) ITC isotherm and thermodynamic fitting for the binding between HA and CB[7] in water. [HA] (cell) = 10 µm; CB[7] (syringe) = 100 µm. (C) Absorption and (D) fluorescence spectra of **GA‐1** (30 µm) upon the sequential addition of CB[7] (60 µm) and HA (60 µm), Ex = 440 nm. (E) ^1^H NMR spectra of **GA‐1**, **CB[7]_2_⊃GA‐1**, and the mixture **CB[7]_2_⊃GA‐1** + HA in D_2_O at 298 K, [**GA‐1**] = 0.5[CB[7]] = 0.5[HA] = 0.5 mm.

The displacement process was initially verified using spectroscopic methods. As shown in Figure 3C, upon the addition of HA to the probe, the absorption spectrum of **CB[7]_2_⊃GA‐1** underwent a blue shift, fully reverting to the characteristic profile of free **GA‐1**. A similar phenomenon was observed in the fluorescence spectra, where the maximum emission wavelength of **GA‐1** blue‐shifted from 560 nm (assembled state) to 500 nm (free state), directly reflecting the guest competition and displacement event (Figure 3D). Conversely, in the absence of CB[7], none of the guest molecules (**GA‐1** to **GA‐3**) exhibited any spectral response to HA (Figure S25), confirming that the interaction between HA and the probe occurs via a host‐guest competitive displacement process within the **CB[7]_2_⊃GA‐1** assembly, rather than through direct covalent or non‐covalent interactions with the dye guest itself. Furthermore, HRMS confirmed the disappearance of the characteristic m/z peak assigned to the supramolecular complex **CB[7]_2_⊃GA‐1**, accompanied by the concurrent reappearance of peaks corresponding to free **GA‐1** (Figure S26). To structurally corroborate the origin of these spectral responses at the molecular level, ^1^H NMR spectroscopy was employed to characterize the probe before and after the addition of HA. As depicted in Figure 3E, following the addition of HA, the proton signals H(1,2) and H(6–8) of the phenyl branches of **GA‐1**—which were originally shifted upfield due to encapsulation—returned to the downfield region, exhibiting chemical shifts nearly identical to those of free **GA‐1**. Furthermore, given the comparable binding constants of CB[7] for the two aromatic branches of the probe and the absence of any observable singly bound **GA‐1**+CB[7] species upon guest competition, we speculate that the competitive binding of HA to the CB[7] moieties on the two branches of the supramolecular probe is more likely to proceed via a random, concurrent process rather than a specific sequential one. Collectively, these results provide conclusive evidence that **GA‐1** is successfully displaced from the macrocyclic cavity by HA due to the superior binding affinity of HA for CB[7], thereby fully accounting for the observed spectral response (Figure 3C,D).

### 2.3 Ratiometric Fluorescence Sensing and the Response Kinetics of the Supramolecular Probe for HA In Vitro

Building on the elucidated response mechanism and promising photophysical properties of the supramolecular probe **CB[7]_2_⊃GA‐1**, its quantitative sensing performance for HA was subsequently evaluated in vitro. Absorption and fluorescence titration experiments were initially conducted by treating **CB[7]_2_⊃GA‐1** with increasing concentrations of HA in cell lysis buffer. As illustrated in Figure 4A, increasing HA concentrations induced the emergence and gradual intensification of a new blue‐shifted absorption peak at 420 nm, accompanied by a concomitant attenuation of absorbance at 455 nm. This spectral evolution approached completion upon the addition of 2 equivalents of HA, yielding an absorption profile consistent with that of the free guest molecule **GA‐1** (Figure 3C). Concurrently, synchronous changes were observed in the fluorescence emission spectra (Figure 4B), where the maximum emission peak underwent a blue shift from 560 to 500 nm, featuring a well‐defined isosbestic point at 520 nm, confirming the conversion of the probe **CB[7]_2_⊃GA‐1** into free **GA‐1**. Linear regression analysis of the dual‐channel fluorescence intensity ratio (F_500 nm_/F_560 nm_) against HA concentration revealed a strong linear relationship (R^2^ = 0.996) over the HA concentration range of 0–40 µm (Figure 4C). The limit of detection (LOD) of the probe **CB[7]_2_⊃GA‐1** for HA was calculated to be 13.2 nm based on the equation LOD = 3*σ*/*k* (where σ is the standard deviation and *k* is the slope of the calibration curve), demonstrating the probe's capability for highly sensitive ratiometric detection of HA.

**FIGURE 4:**
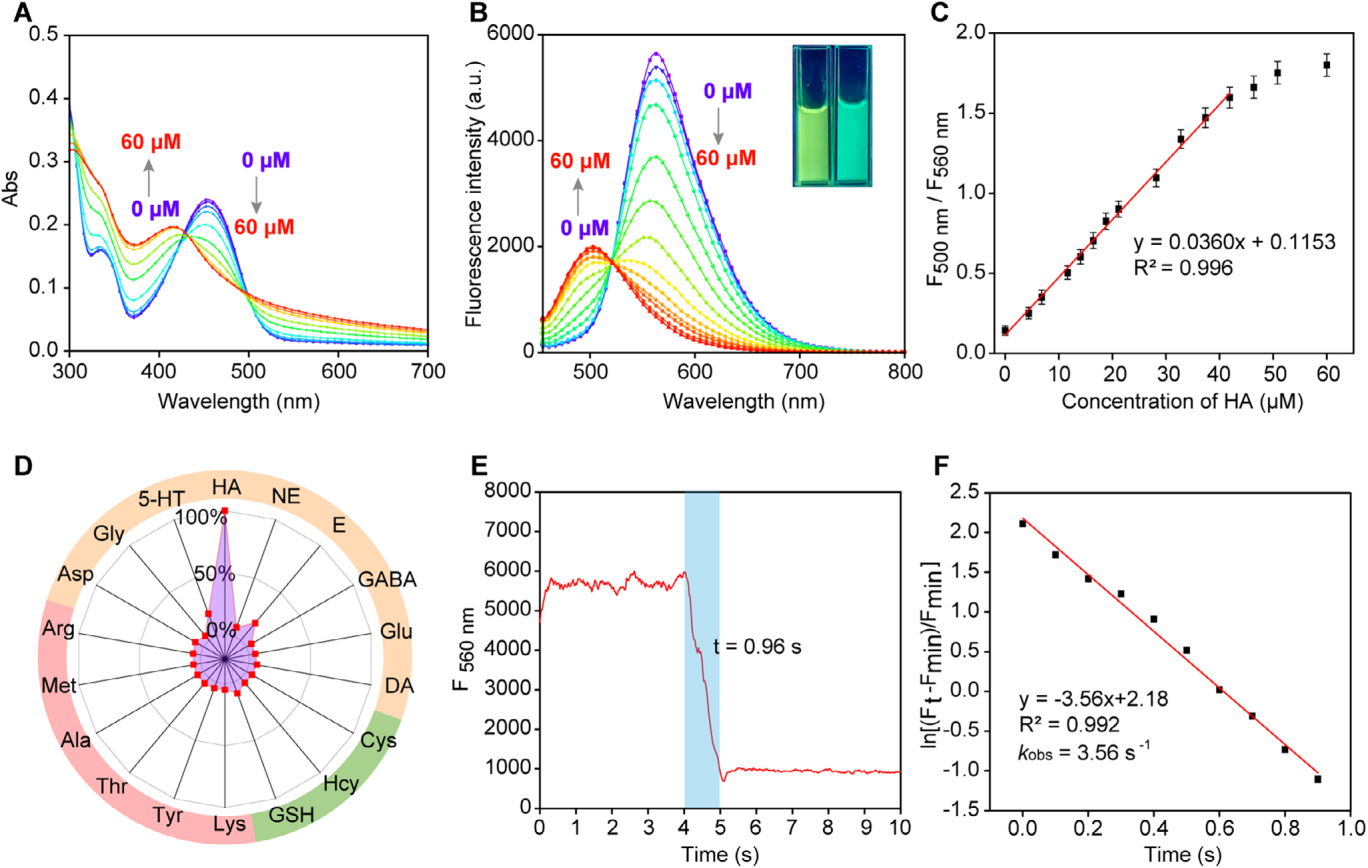
Sensing performance of probe **CB[7]_2_⊃GA‐1** toward HA. (A) Absorption and (B) fluorescence emission spectral changes of **CB[7]_2_⊃GA‐1** (30 µm) upon titration with HA (0–60 µm) in cell lysis buffer (10 mm, pH 7.4, containing 1% DMSO), Ex = 440 nm. Inset: Photographs of the **CB[7]_2_⊃GA‐1** solution under 365 nm UV illumination before (left) and after (right) the addition of HA. (C) Plot of the fluorescence intensity ratio of **CB[7]_2_⊃GA‐1** versus HA concentration (0–60 µm). Data are presented as mean ± S.D. (*n* = 3 independent experiments). (D) Selectivity of **CB[7]_2_⊃GA‐1** toward various analytes in cell lysis buffer. The response is represented as the relative fluorescence ratio (F_500 nm_/F_560 nm_) upon exposure to HA (set as 100%), 5‐hydroxytryptamine (5‐HT), norepinephrine (NE), epinephrine (E), dopamine (DA), γ‐aminobutyric acid (GABA), glutamic acid (Glu), cysteine (Cys), homocysteine (Hcy), lysine (Lys), tyrosine (Tyr), threonine (Thr), alanine (Ala), methionine (Met), arginine (Arg), aspartic acid (Asp), and glycine (Gly) at 100 µm, and glutathione (GSH) at 1 mm. Data are presented as mean ± S.D. (*n* = 3). (E) Fluorescence response kinetics at 560 nm (Ex = 440 nm) for **CB[7]_2_⊃GA‐1** (30 µm) upon the addition of HA (60 µm) in cell lysis buffer. (F) Exponential fitting of the fluorescence time‐traces for **CB[7]_2_⊃GA‐1** (30 µm) following the addition of HA (60 µm) to determine the observed rate constant (*k*_obs_).

Selectivity is a critical parameter governing the applicability of a probe within complex biological environments. Accordingly, we systematically assessed the selectivity and anti‐interference capability of **CB[7]_2_⊃GA‐1** toward various neurotransmitters, amino acids, biothiols, polyamines, etc. As shown in Figure 4D, using the HA‐induced fluorescence ratio (F_500 nm_/F_560 nm_) as the reference (100%), the relative response signals elicited by all other potential interferents remained below 20%, underscoring the exceptional selectivity of **CB[7]_2_⊃GA‐1** for HA. Furthermore, interference assays demonstrated that systems containing HA alongside various coexisting analytes (Figures S27 and S28) yielded fluorescence signals nearly identical to those of HA alone, confirming that these interferents do not significantly compromise HA detection. Given the rapid release kinetics characteristic of HA, the development of fast‐responsive probes is paramount. We therefore evaluated the probe's response rate using a stopped‐flow rapid mixing apparatus (Figure S29). Figure 4E depicts the fluorescence response kinetics monitored at 560 nm (excitation at 440 nm). The probe exhibited an extremely rapid response to HA within 0.96 s. Subsequent kinetic fitting yielded an apparent rate constant (*k*_obs_) of 3.56 s^−^^1^ (Figure 4F), highlighting the ultrafast kinetic characteristics of the supramolecular interaction between HA and the probe, which ensures prompt responsiveness for HA detection. To further evaluate the practical applicability of the probe, we examined the pH stability of both **CB[7]_2_⊃GA‐1** and its recognition product with HA. As depicted in Figure S30, the fluorescence intensity ratio (F_500 nm_/F_560 nm_) for both solutions remained stable across a pH range of 3–8, ensuring reliable detection performance under varying physiological conditions. Additionally, prolonged continuous illumination experiments (2.5 h) revealed negligible fluctuations in fluorescence intensity for both systems throughout the test period (Figure S31), demonstrating that both the probe and its HA‐induced product possess excellent photostability, rendering them highly suitable for long‐term bioimaging applications.

### 2.4 Ratiometric Fluorescence Imaging of HA Dynamics in Living Mast Cells During Degranulation

In this section, the performance of the supramolecular probe **CB[7]_2_⊃GA‐1** for sensing and imaging HA was further evaluated in living systems. Initially, a cellular uptake assay was conducted to characterize the cellular internalization efficiency of the supramolecular probe, which demonstrated efficient cellular uptake (Figure S32). Subsequently, the cytotoxicity of the probe was assessed using a standard methyl thiazolyl tetrazolium (MTT) assay [41, 42]. As shown in Figure S33, the viability of mast cells remained above 90% following treatment with varying concentrations of **CB[7]_2_⊃GA‐1** (0–50 µm) for both 12 and 24 h. Furthermore, the biocompatibility of the probe was examined via flow cytometry using an Annexin V‐FITC apoptosis kit [43, 44]. As illustrated in Figure S34, mast cells incubated with different concentrations of **CB[7]_2_⊃GA‐1** (0, 10, 20, and 30 µm) exhibited no significant difference in viability compared to the control group. Moreover, intracellular stability and competition assays confirmed that the fluorescence signal of probe‐stained cells remained stable over 36 h, and that relevant small‐molecule interferences at physiological concentrations were insufficient to perturb the luminescence of the supramolecular probe in cells (Figures S35 and S36). These results demonstrate that the probe possesses low cytotoxicity and excellent biocompatibility and stability, rendering it suitable for HA detection in living systems. Subsequently, the subcellular localization of the probe was investigated. Co‐localization experiments were performed by co‐incubating mast cells with **CB[7]_2_⊃GA‐1** and a commercial cytoplasmic marker (Alexa Fluor 660). Confocal fluorescence microscopy images collected in the green channel (**CB[7]_2_⊃GA‐1**) and the red channel (Alexa Fluor 660) revealed a strong overlap between the two emission signals (Pearson's correlation coefficient, ρ = 0.93; Figure S37). This indicates that **CB[7]_2_⊃GA‐1** is membrane‐permeable and localizes primarily to the cytoplasm, thereby enabling effective intracellular detection and imaging.

Building upon these results, we further evaluated the capability of probe **CB[7]_2_⊃GA‐1** to monitor both exogenous and endogenous HA dynamics in mast cells in real time. As depicted in Figure S38, mast cells preincubated with **CB[7]_2_⊃GA‐1** displayed intense luminescence at 560 nm (pseudo‐colored red) and weak emission at 500 nm (pseudo‐colored green). Upon the addition of exogenous HA (0–40 µm), a marked decrease in 560 nm luminescence accompanied by a concurrent increase in 500 nm emission was observed, yielding a distinct ratiometric response to HA, with the F_500 nm_/F_560 nm_ ratio increasing from 0.12 to 1.55. Endogenous HA is typically synthesized and sequestered within secretory granules. Under conditions of physiological impairment—such as lysosomal dysfunction or drug‐induced toxicity—granular HA is released into the cytoplasm or the extracellular space. Monensin and nigericin are ionophores that facilitate the rapid exchange of protons from secretory granules with extracellular or cytoplasmic Na^+^/K^+^, inducing a swift intragranular pH shift and the consequent release of HA into the cytoplasm [45, 46]. Therefore, we employed monensin and nigericin as pharmacological stimuli to investigate endogenous HA dynamics in mast cells. As illustrated in Figure S38, stimulation of probe‐preincubated mast cells with 10 µm monensin or nigericin resulted in a significant increase in the fluorescence intensity ratio (F_500 nm_/F_560 nm_), indicating drug‐triggered HA release from secretory granules into the cytoplasm. Collectively, these findings demonstrate that the probe enables the quantitative detection of both exogenous and endogenous HA in living cells.

Based on the aforementioned feasibility studies, we further evaluated the real‐time dynamic imaging performance of probe **CB[7]_2_⊃GA‐1** for HA in living cells. Conventional detection methods, limited by their slow response times, are restricted to measuring HA release under static conditions. Consequently, they fail to capture rapid fluctuations in cytoplasmic HA concentrations and struggle to achieve in situ, single‐cell detection. In contrast, the **CB[7]_2_⊃GA‐1** probe developed in this work features a sub‐second response time, offering a distinct advantage for real‐time imaging. We therefore applied it to dynamically monitor HA levels in mast cells under pharmacological stimulation. As shown in Figure 5A,B, equal concentrations (15 µm) of monensin or nigericin were added in parallel to mast cells preincubated with **CB[7]_2_⊃GA‐1** (30 µm). Confocal fluorescence imaging was immediately initiated and continued for 10 min (1 frame per second), allowing real‐time recording of time‐dependent fluorescence intensity changes in each channel. The results revealed that upon the addition of either drug, the ratiometric fluorescence signal (F_500 nm_/F_560 nm_) increased markedly within a short timeframe, indicating a rapid and sustained rise in cytoplasmic HA concentration following stimulation. Furthermore, at identical drug concentrations, nigericin induced a faster ratiometric change (reaching saturation in 250 s) compared to monensin (350 s). Moreover, the magnitude of the fluorescence ratio increase triggered by nigericin exceeded that of monensin, demonstrating that nigericin induces a more rapid and substantial leakage of HA from secretory granules into the cytoplasm. This is because nigericin as a K^+^/H^+^ exchanger, efficiently mediates the exchange of intragranular H^+^ for extracellular K^+^, leading to rapid alkalinization of the granule interior. This swift disruption of the granular pH homeostasis triggers immediate degranulation and a fast release of HA [46]. In contrast, monensin primarily mediates Na^+^/H^+^ exchange, which proceeds at a relatively slower ion transport rate and perturbs the granular microenvironment more mildly, thus resulting in a more gradual and delayed histamine release profile [10]. Therefore, these findings provide precise spatiotemporal molecular data for investigating HA‐related mast cell pathologies.

**FIGURE 5:**
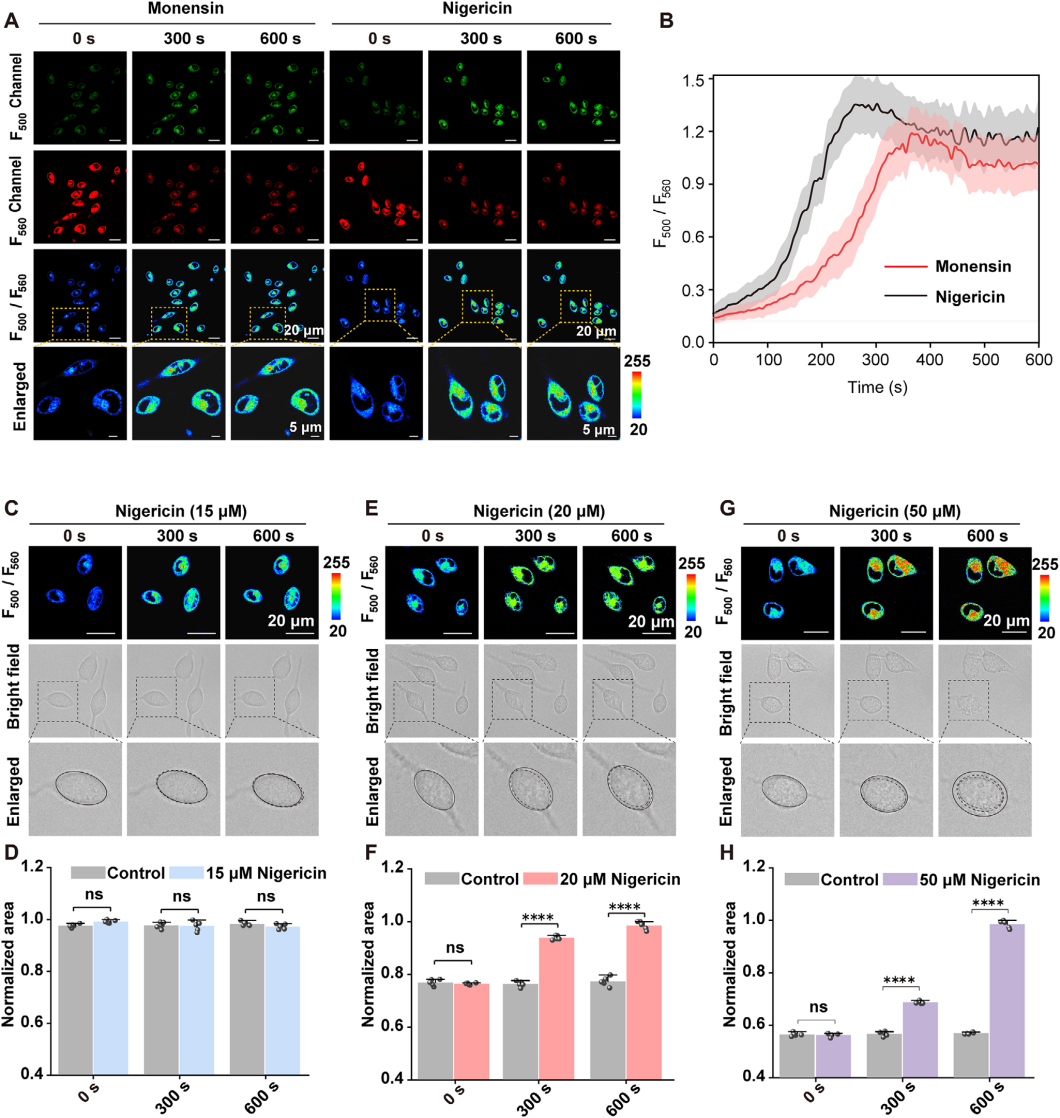
Real‐time ratiometric fluorescence imaging of HA in mast cells under storage dysfunction model. (A) Fluorescence images of mast cells preincubated with the probe CB[7]_2_⊃GA‐1 (30 µm) and subsequently stimulated with monensin and nigericin (15 µm) over a period of 600 s. Ex = 440 nm. (B) Time course of the fluorescence ratio (F_500 nm_/F_560 nm_) under the stimulation conditions described in (A). (C, E, G) Fluorescence ratio F_500 nm_/F_560 nm_ images and (D, F, H) the corresponding changes in cell volume for mast cells preincubated with CB[7]_2_⊃GA‐1 (30 µm) followed by stimulation with nigericin at concentrations of 15 µm (C, D), 20 µm (E, F), and 50 µm (G, H). Ex = 440 nm. Statistical significance of cell volume changes relative to the control group was determined using Student's *t*‐test. ns, no significant difference; ^****^ *p* < 0.0001. Data are presented as mean ± S.D. (*n* = 5). Scale bars: 20 µm (global images) and 5 µm (enlarged images).

As previously reported, the impairment of the HA storage function in mast cells may lead to intracellular granule swelling, resulting in increased cell volume and significant morphological alterations [47]. To comprehensively investigate and visualize this storage dysfunction, we incubated mast cells with varying concentrations of nigericin (15, 20, and 50 µm) and monitored changes in both HA concentration and cellular morphology. Here, cell boundaries were manually delineated from bright‐field images, and the resulting 2D projected cell area was quantified using ImageJ [48, 49, 50]. As illustrated in Figure 5C–H, stimulation with different drug concentrations induced marked differences in both the fluorescence ratio (F_500 nm_/F_560 nm_) and mast cell morphology. Specifically, within the studied concentration range, cytoplasmic HA content exhibited a positive correlation with nigericin concentration. Furthermore, bright‐field imaging revealed a concentration‐dependent relationship between mast cell size and nigericin exposure: at 15 µm, cell morphology remained largely unchanged compared to the control group, despite an 18‐fold increase in HA concentration (Figure 5C,D); at 20 µm, cell volume progressively increased over time relative to the control, accompanied by a 20‐fold rise in HA concentration (Figure 5E,F); at 50 µm, HA levels increased by 26‐fold within 600 s, and pronounced cell swelling was observed, ultimately leading to membrane rupture and the leakage of intracellular contents (Figure 5G,H). Collectively, these results demonstrate a positive correlation among nigericin dosage, intracellular HA content, and mast cell morphological status (specifically, cell volume expansion). This suggests that the probe developed in this study can serve as a quantitative fluorescent indicator for evaluating and providing early warnings regarding the progression of drug‐induced storage dysfunction in mast cells. Thus, it provides a foundation for the early detection of mast cell‐related pathological processes, as well as informing future drug development and therapeutic interventions.

## 3 Conclusion

In summary, we have engineered a supramolecular ratiometric fluorescent probe, **CB[7]_2_⊃GA‐1**, designed for the rapid and sensitive detection of HA. Through the strategic modulation of the cationic center's position on the guest molecule, we achieved precise control over the self‐assembly stoichiometry with CB[7]. Specifically, the centrally positioned positive charge in **GA‐1** facilitates the formation of a sterically unhindered, stable 1:2 complex, activating a long‐range ICT mechanism that results in a pronounced red shift in fluorescence. Operating via a competitive displacement mechanism, the probe exhibits state‐of‐the‐art performance metrics, including sub‐second response kinetics (0.96 s), high sensitivity (LOD = 13.2 nm), superior selectivity against biological interferents, and a robust ratiometric fluorescence readout. These attributes enable the precise, real‐time monitoring of HA dynamics that remain inaccessible to conventional reaction‐based probes. We successfully applied this system to visualize both exogenous HA uptake and endogenous HA release triggered by monensin or nigericin in living mast cells. Notably, the probe captured the concentration‐ and time‐dependent leakage of HA during drug‐induced storage dysfunction, revealing a positive correlation among drug dosage, cytoplasmic HA levels, and cell volume expansion culminating in membrane rupture. This work not only establishes a straightforward and precise supramolecular structural regulation strategy to achieve rapid ratiometric responses toward small‐molecule neurotransmitters, but also provides a quantitative link between subcellular analyte dynamics and the morphological evolution of mast cell dysfunction, thereby opening new avenues for the early diagnosis and therapeutic assessment of associated pathological conditions.

## Author Contributions

**Chenming Chan**: conceptualization, methodology, investigation, validation, visualization, Writing – original draft, formal analysis, data curation. **Yujie Han**: formal analysis, visualization. **Yu‐Fei Song**: formal analysis, visualization. **Chunlei Jiang**: formal analysis, visualization. **Qi‐Wei Zhang**: conceptualization, validation, formal analysis, supervision, project administration, funding acquisition, writing – review and editing.

## Conflicts of Interest

The authors declare no conflicts of interest.

## Supporting information




**Supporting File**: advs78254‐sup‐0001‐SuppMat.pdf.

## Data Availability

The data that support the findings of this study are available in the Supporting Information of this article.
