# Supplementary material for: Sub‐Second Ratiometric Fluorescence Imaging of Histamine Dynamics in Living Cells via a Supramolecular Probe
Source: Adv Sci (Weinh). 2026 Oct 14:e78254. Online ahead of print. doi: 10.1002/advs.78254 (PMC13669221; doi:10.1002/advs.78254)
Supplement: Supplementary file 1 [file ADVS-9999-e78254-s001.pdf]

neuornSupporting Information

Sub-Second Ratiometric Fluorescence Imaging of Histamine Dynamics in Living Cells via a Supramolecular Probe

Chenming Chan,[1] Yujie Han,[1] Yu-Fei Song,[1] Chunlei Jiang,[1] and Qi-Wei Zhang[1,2]*

[1] School of Chemistry and Molecular Engineering, East China Normal University, Dongchuan Road 500, Shanghai 200241, China.

[2] Institute of Magnetic Resonance and Molecular Imaging in Medicine, East China Normal University, Dongchuan Road 500, Shanghai 200241, China.

***Corresponding Authors:**

qwzhang@chem.ecnu.edu.cn (Q.-W. Z.)

Table of contents

1. Materials and methods 2

2 Syntheses and structural characterizations 6

3. Optical measurements and biological experiments. 17

4. References 27

1. Materials and methods

All chemicals and reagents were obtained from commercial sources and utilized without additional purification. Distilled water was employed after treatment with an ultra-purification system. Solvents of analytical grade and all reagents were sourced from Sinopharm Chemical Reagent Co. Ltd. (Shanghai, China). 3-(4,5-Dimethyl-2-Thiazolyl)-2,5-Diphenyl Tetrazolium Bromide (MTT) was obtained from Sigma-Aldrich (U.S.A.).

1.1 Determination of the fluorescence quantum yields (Φ)

Rhodamine 6G is selected as a reference standard (Φ = 0.95 in ethanol). The quantum yields of the samples are calculated using the following formula:

$$\Phi_{sample} = \Phi_{std} \cdot (I_{sample} \cdot \mathrm{A}_{\mathrm{std}}) / (I_{std} \cdot A_{sample}) \cdot (n_{sample} / n_{std})^2$$

Φ_{sample} and Φ_{std} represent the quantum yields of the sample and the standard, respectively. I_{sample} and I_{std} refer to the integrated areas under the emission spectra for the sample and standard. A_{sample} and A_{std} indicate the absorbance at the excitation wavelength, while n is the refractive index of the solution used.

1.2 Calculations of the detection limit (LOD)

The limit of detection (LOD) is determined from fluorescence titration experiments. This calculation is derived from a plot of the ratio of fluorescence intensity versus HA concentration:

$$\mathrm{LOD} = 3\sigma / k$$

where σ is the standard deviation of sample, and k is the slope of the calibration curve.

1.3 Instruments.

^{1}H NMR, ^{13}C NMR, and relevant 2D NMR spectra were measured on a Bruker AVANCE III 600 MHz spectrometer (Germany). High-resolution mass spectrometry was performed on a Bruker ESI time-of-flight mass spectrometer and a Thermo Scientific Q Exactive Instrument (Germany). The absorption spectra were recorded in a rectangular quartz cell (10 × 10 × 45 mm) on the Shimadzu UV-1900i spectrometer (Japan). The fluorescence spectroscopy and fluorescence kinetics

studies were performed using Hitachi F-4600 fluorescence spectrometer (Japan). The rapid fluorescence response of the probe to HA was analyzed using a stopped-flow accessory with a pneumatic drive system (SFA-20, HI-Tech, TgK Scientific, United Kingdom). The pH measurements were conducted with a digital pH meter (pH-3c, Shanghai Lei Ci Device Works, China) containing a composite glass electrode. The Leica Stellaris 8 Sted (an inverted fluorescence microscope) equipped with an oil immersion objective (63×) was used for fluorescence confocal imaging. FACS Calibur flow cytometry (Becton, Dickinson and Company, USA) was used for cell apoptosis assay. All tests were performed at 298 K unless specifically mentioned.

1.4 Kinetics study procedures

The rapid fluorescence response of the probe **$CB[7]_2$⊃GA-1** to HA was analyzed using a stopped-flow accessory with a pneumatic drive system. Equal volumes of the probe solution (60 μM, 150 μL, syringe A) and pure water (150 μL, syringe B) were rapidly driven from syringes into a highly efficient mixer and the basal fluorescence intensity of the probe was monitored at 560 nm. Subsequently, HA (120 μM, 150 μL, syringe B') and the probe (60 μM, 150 μL, syringe A) solutions were rapidly driven from both syringes to the mixer in the same way to initiate the fast reaction. The resultant reaction volume then displaced the contents of the optical cell, thus filling it with freshly mixed reagents. During the entire mixing process, the fluorescence intensity was continuously monitored. All the injected volume was limited by a stop syringe that provided the "stopped-flow". The dead time of such mixing system was ca. 8 ms, and the fluorometer has a shorter sampling interval (5 ms).

1.5 Theoretical Calculations.

The DFT calculations for CB[7]⊃HA were conducted based on the Vienna Ab-initio Simulation Package (VASP)[1-2]. The exchange-correlation effects were described by the Perdew-Burke-Ernzerhof (PBE) functional within the generalized gradient approximation (GGA) method[3-4]. The core-valence interactions were accounted for by the projector augmented-wave (PAW) method[5]. The energy cutoff for plane wave expansions was set to 480 eV, and the 3×3×1 Monkhorst-Pack grid k-points were selected to sample the Brillouin zone integration. A vacuum space of 15 Å was adopted

to avoid periodic interactions. The structural optimization was completed for energy and force convergence set at 1.0×10^{-4} eV and 0.02 eV $Å^{-1}$, respectively.

The binding energy can be calculated according to the following formula:

$$E_{bind} = E(A+B) - E(A) - E(B)$$

where E_{bind} represents the binding energy, E(A+B) is the calculated total energy of the combined system, E(A) and E(B) mean the calculated energy of component A and component B, respectively.

The calculations for the structural optimization section in this work were performed using Gaussian 16 program package. Full geometry optimizations were performed to locate all the stationary points, using the B3LYP[6] with the 6-31G(d)[7]. Dispersion corrections were computed with Grimme's D3(BJ) method in optimization[8]. Convergence parameters of the default threshold were retained (maximum force within 4.5×10^{-4} Hartrees/Bohr and root mean square (RMS) force within 3.0×10^{-4} Hartrees/Radian) to obtain the optimized structure. Vertical excitations were then calculated at the TD-B3LYP/6-31G(d) level to obtain the energy levels of both singlet and triplet excited states.

1.6 Cell culture and confocal imaging.

The mast cells (Cell line: RBL-2H3, RRID: CVCL_0591)) were obtained from Wuhan Pricella Biotechnology Co., Ltd. The cells were seeded into a 6-well cell culture plate in DMEM (Dulbecco's modified Eagle's medium) supplemented with 10% FBS (fetal bovine serum) and 1% PS (Penicillin-Streptomycin) at 37°C under an atmosphere of 5% CO_2. The medium was changed every 2–3 days. Confocal imaging of probe-stained cells was performed with an excitation wavelength of 440 nm. The emission was collected in two channels: 490–510 nm for the F_{500} channel and 550–570 nm for the F_{560} channel.

1.7 Apoptosis assay and cytotoxicity analysis.

For the apoptosis assay, mast cells were cultured with different concentrations of probe **CB[7]$_2$⊃GA-1** (0, 10, 20, 30 μM) for 24 h. After removing the culture media, the cells were collected with the help of EDTA-free trypsin. After washing with PBS (1 mL × 3 times), the cells were resuspended in 300 μL of binding buffer and further incubated with FITC-Annexin V and PI

to label the apoptosis cells and necrotic cells, respectively. For the cytotoxicity analysis, various concentrations (0, 5, 10, 20, 30, 40 and 50 μM) of **CB[7]$_2$⊃GA-1** were added to the preincubated mast cells in 96-well plates and cultured for 12 and 24 h. The original medium was exchanged followed by the addition of 10 μL MTT (0.5 mg mL^{-1}). Four hours later, the medium was removed, and 200 μL DMSO was added to each well. The absorbance at 490 nm was measured by using a Spectramax microwell plate reader, and the cell viability towards the cell line was measured using the given equation:

Cell viability (%) = Mean absorbance (Treated cell) / Mean absorbance (Control cell) × 100%

1.8 General information of previously reported fluorescent probes for histamine detection.

Table S1. Comparison of previously reported small-molecule fluorescent probes for HA sensing.

Reference	Emission Wavelength	Detection mode	Detection range	Response time	Application
Chem. Eur. J. 2017, 23, 11891-11897.	514 nm	Turn-off	0- 0.12 mM	30 min	Water, human serum, urine, cells
Anal. Chim. Acta 2015, 880, 145-151.	475 nm	Turn-on	0.2-4 mM	60 min	Soy sauce
Anal. Biochem. 2010, 404, 135-139.	535 nm	Turn-on	0-1 mM	Not available	RAW264 cells
Anal. Chem. 2016, 88, 1526-1529.	685 nm	Turn-on	0-2 mM	3 min	Mast cells
Chem. Commun., 2012, 48, 7401-7403.	432 nm	Turn-on	Not available	30 min	RAW264 cells, RBL-2H3 cells
This work	500 nm/560 nm	ratiometric	0-40 μM	0.96 s	Mast cells

2 Syntheses and structural characterizations

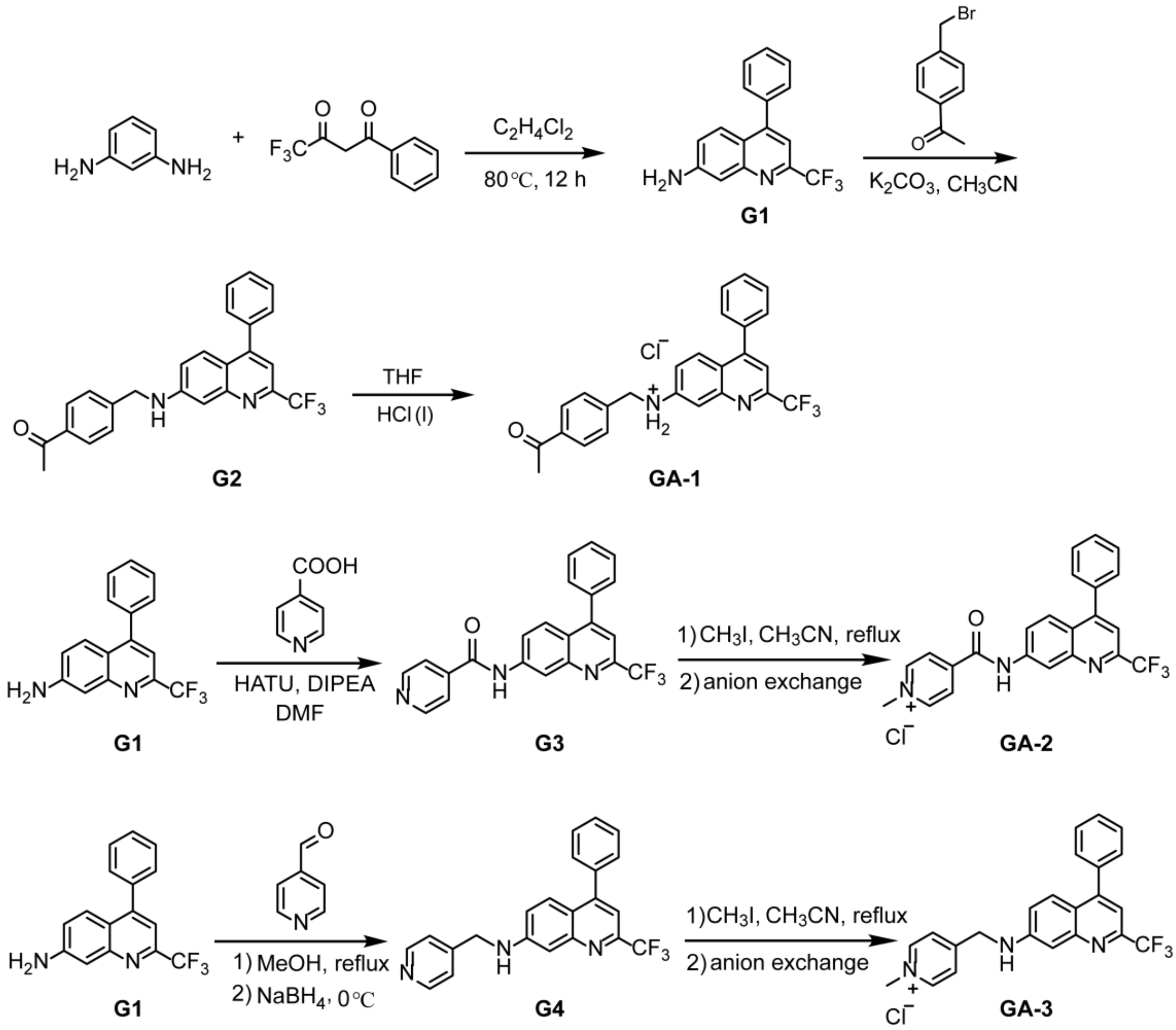

Figure S1. Synthesis route of the guest molecules.

Synthesis of 4-phenyl-2-(trifluoromethyl)quinolin-7-amine (G1)

The mixture of m-phenylenediamine (3.25 g, 30 mmol) and benzoyltrifluoroacetone (6.48 g, 30 mmol) in 1,2-dichloroethane (100 mL) was heated at reflux under nitrogen atmosphere for 12 h. After confirming complete consumption of the starting materials, the reaction mixture was cooled to room temperature and the solvent was removed under reduced pressure. The crude product was purified by silica gel column chromatography using dichloromethane/petroleum ether (1/1, v/v) as eluent to afford compound **G1** as yellow solid (6 g, 69% yield). ^{1}H NMR (600 MHz, DMSO-d_6) δ 8.27 (d, J = 7.2 Hz, 2H), 7.96 (s, 1H), 7.81 (d, J = 8.9 Hz, 1H), 7.55 (dt, J = 23.3, 7.1 Hz, 3H), 7.24-7.16 (m, 2H), 6.16 (s, 2H). ^{13}C NMR (151 MHz, DMSO-d_6) δ 155.51, 151.14, 150.86, 138.11, 129.81, 128.84, 127.14, 123.82, 120.57, 113.02, 110.27, 107.24. HR-MS (ESI) m/z for: $C_{16}H_{12}F_3N_2$ $[M+H]^+$: calcd.289.0953, found: 289.0945.

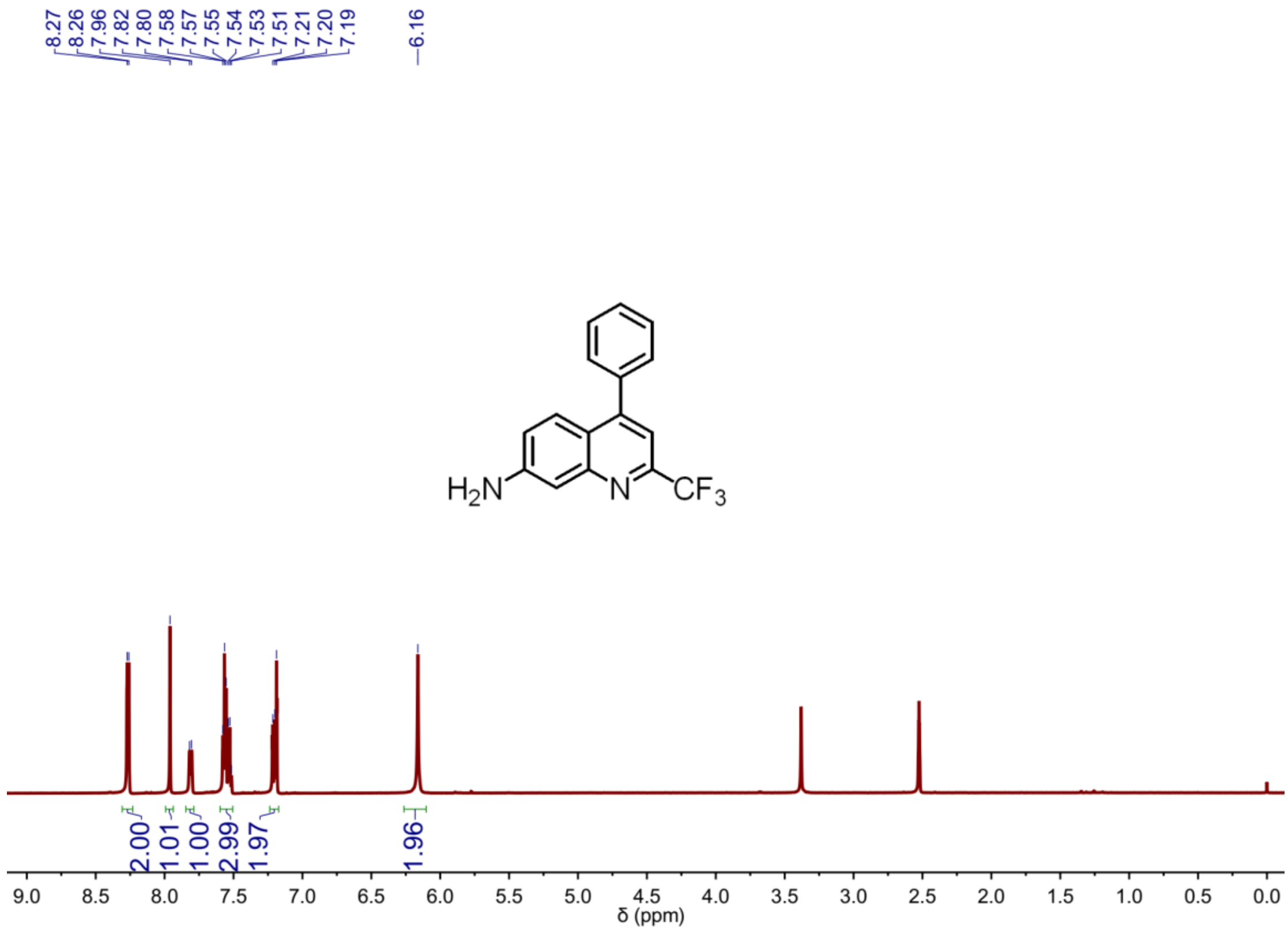

Figure S2. ^{1}H NMR spectrum of **G1** in DMSO-d_6 (25 °C).

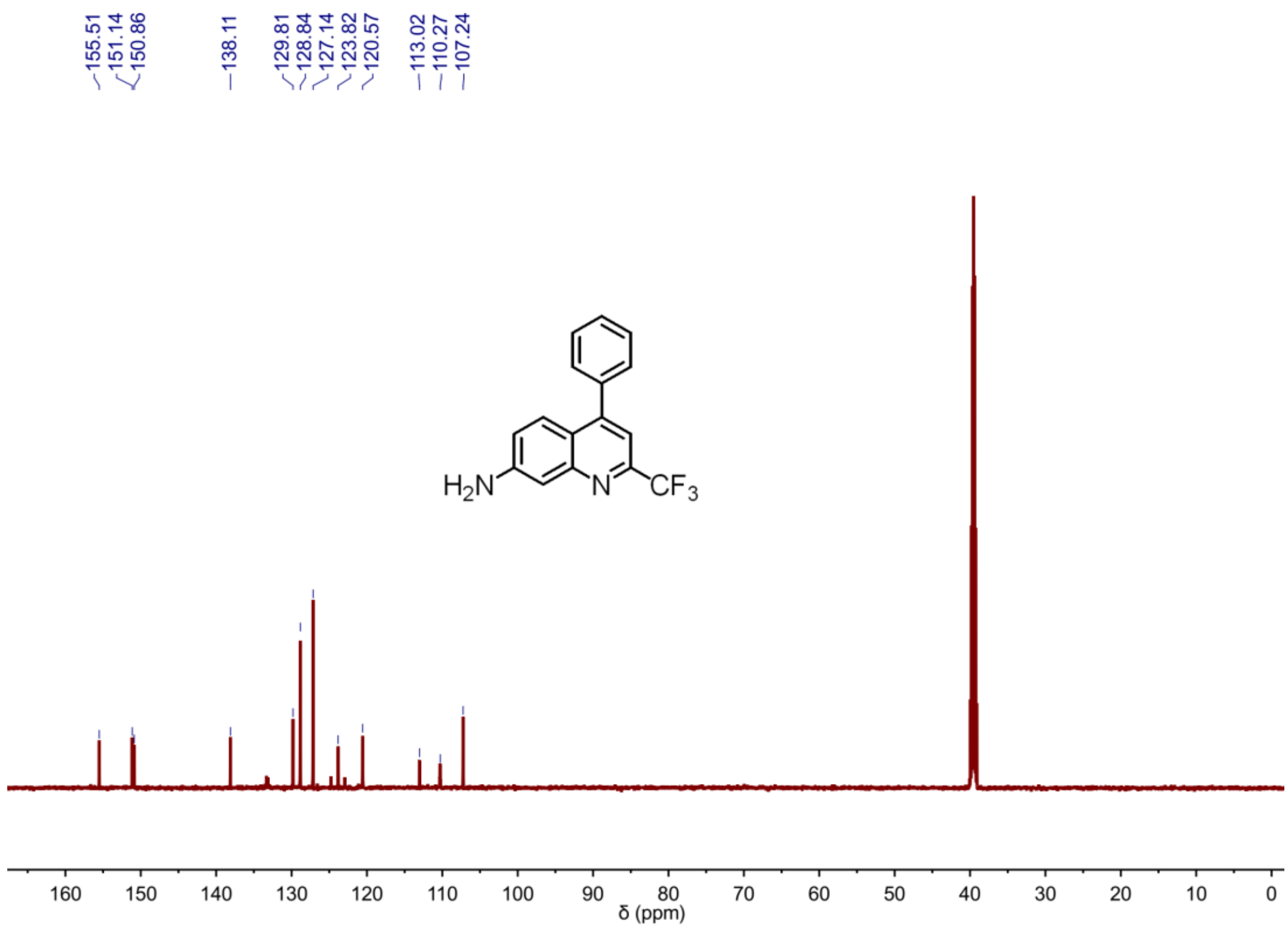

Figure S3. ^{13}C NMR spectrum of compound **G1** in DMSO-d_6 (25 °C).

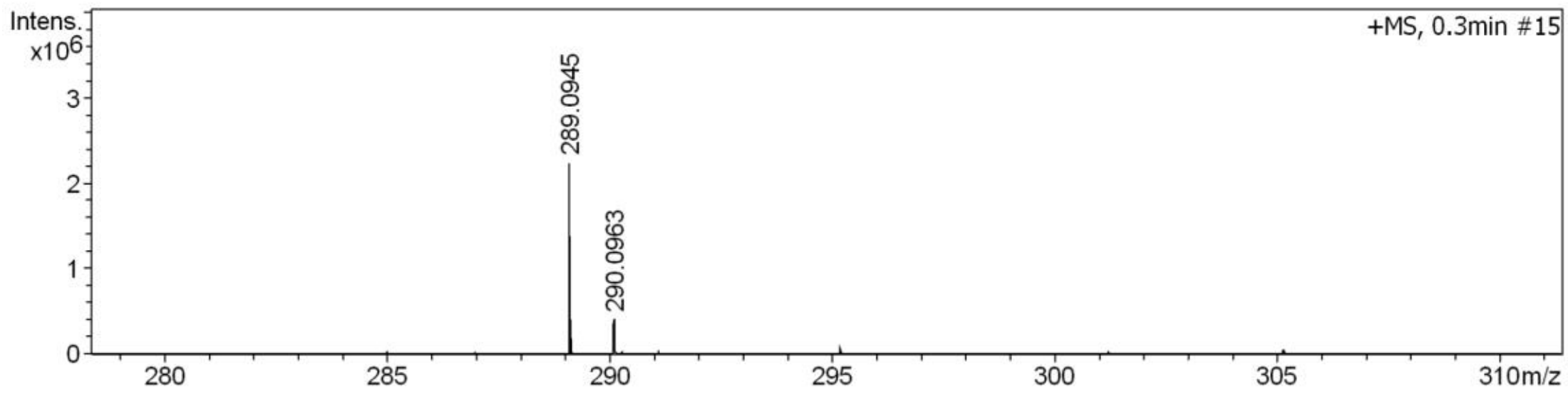

#	m/z	Res.	S/N	I	I %	FWHM
1	289.0945	22679	1748.8	2234804	100.0	0.0127
2	290.0963	23880	319.5	408692	18.3	0.0121
3	291.1002	19902	24.9	31864	1.4	0.0146

Meas. m/z	#	Ion Formula	m/z	err [ppm]	mSigma		Score	rdb	e⁻ Conf	N-Rule
289.0945	1	C16H12F3N2	289.0947	0.7	1.0	1	100.00	10.5	even	ok

Figure S4. ESI-HRMS spectrum of compound **G1**.

Synthesis of 1-(4-(((4-phenyl-2-(trifluoromethyl)quinolin-7-yl)amino)methyl)phenyl)ethan-1-one (G2)

The mixture of compound **G1** (1.26 g, 4.40 mmol) and K_2CO_3 (0.60 g, 4.40 mmol) in acetonitrile (30 mL) under nitrogen atmosphere was heated with stirring until complete dissolution. Then 4-acetylbenzyl bromide (0.94 g, 4.40 mmol) was added, and the resulting mixture was heated at reflux for an additional 12 h. The solvent was removed under reduced pressure. The crude product was purified by silica gel column chromatography using dichloromethane as eluent to give an oily product, which was triturated with petroleum ether to afford compound **G2** as pale-yellow solid (1.80 g, 97% yield). ^1H NMR (600 MHz, DMSO-d_6) δ 8.21 (d, J = 6.5 Hz, 2H), 7.98-7.92 (m, 3H), 7.82 (d, J = 9.1 Hz, 1H), 7.57 (d, J = 8.2 Hz, 2H), 7.55-7.48 (m, 3H), 7.33 (d, J = 9.2 Hz, 1H), 6.91 (d, J = 2.2 Hz, 1H), 4.59 (d, J = 6.0 Hz, 2H), 3.31 (s, 1H), 2.55 (s, 3H). ^{13}C NMR (151 MHz, DMSO-d_6) δ 197.39, 155.55, 150.86, 150.08, 144.88, 137.86, 135.60, 129.78, 129.27, 128.76, 128.44, 127.08, 123.60, 120.69, 113.40, 110.52, 104.50, 45.64, 26.60. HR-MS (ESI) m/z for $C_{25}H_{19}F_3N_2NaO$ $[M+Na]^+$: calcd.443.1342, found: 443.1346.

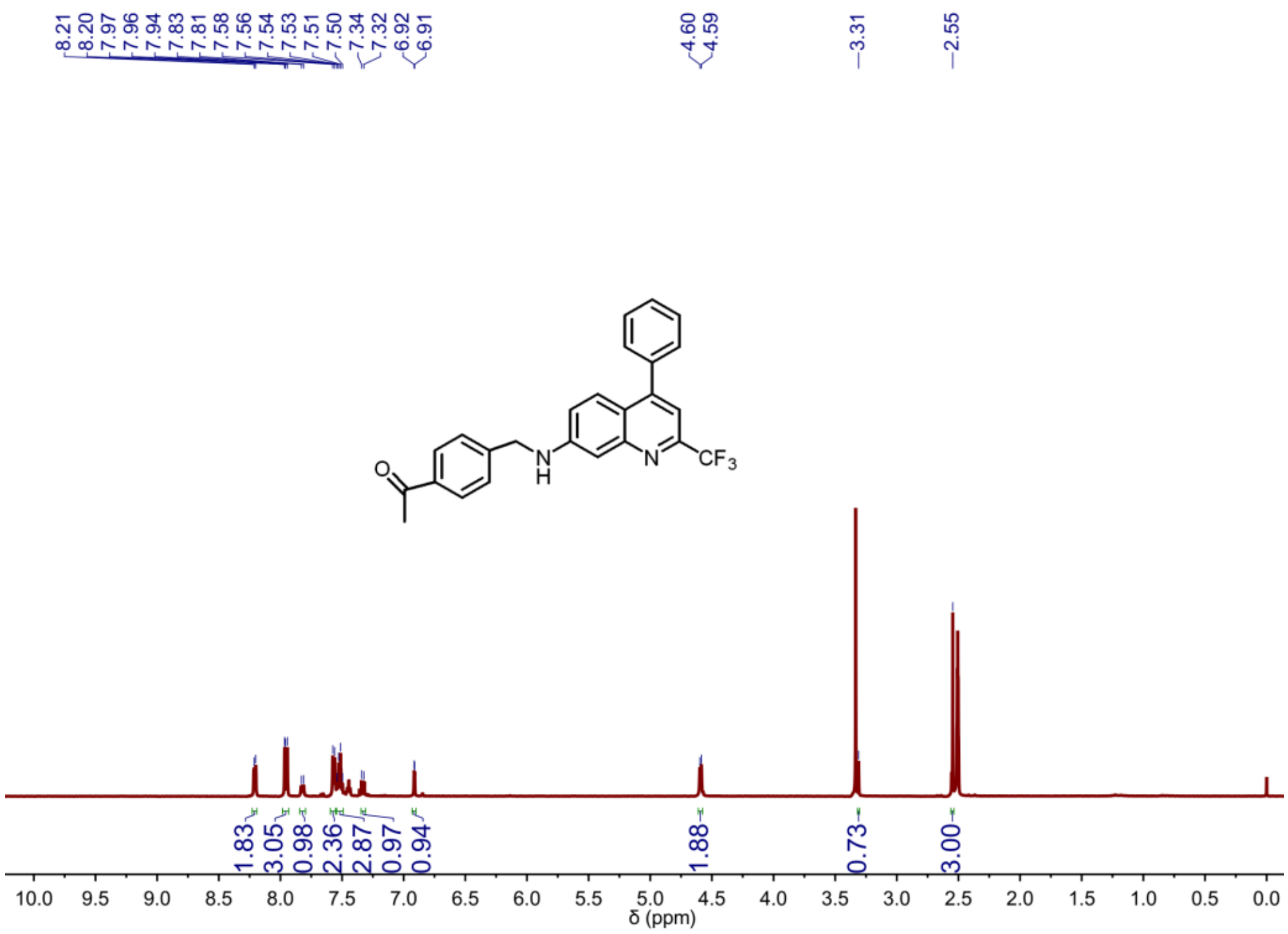

Figure S5. ^{1}H NMR spectrum of **G2** in DMSO-d_6 (25 °C).

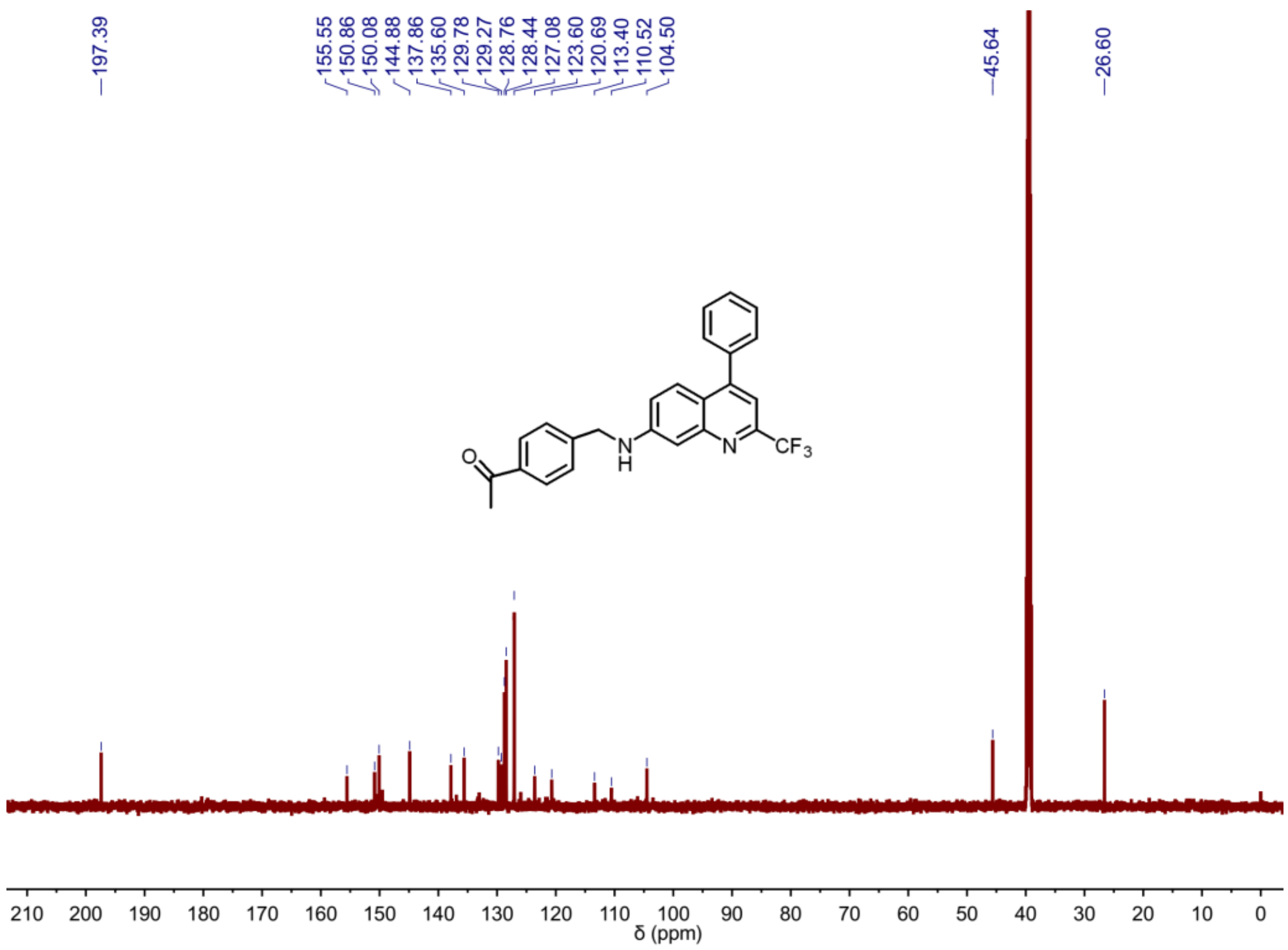

Figure S6. ^{13}C NMR spectrum of compound **G2** in DMSO-d_6 (25 °C).

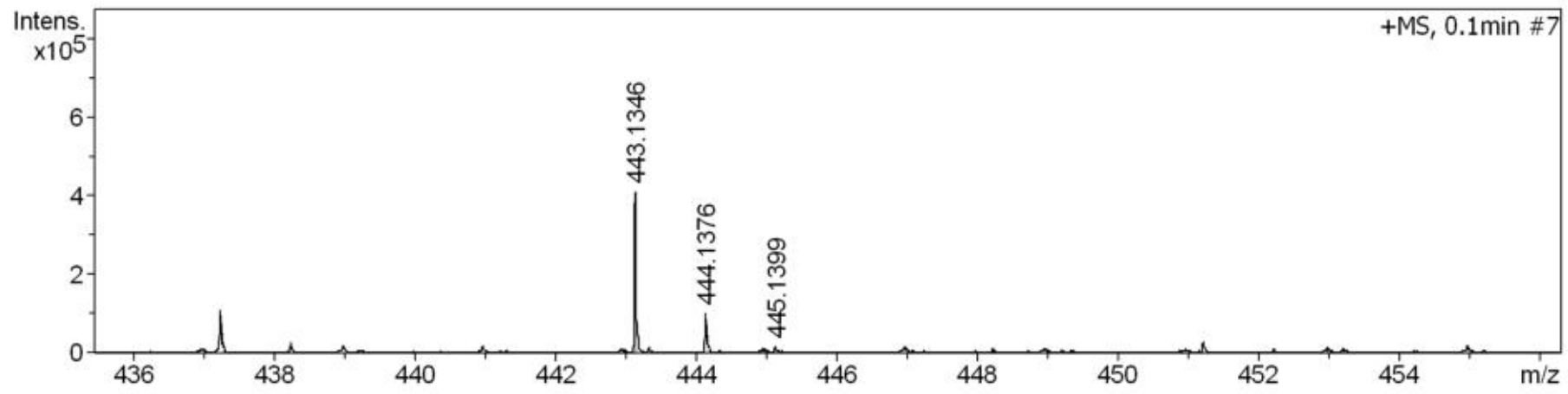

#	m/z	Res.	S/N	I	I %	FWHM
1	443.1346	25743	444.1	410064	100.0	0.0172
2	444.1376	19324	108.9	100792	24.6	0.0230
3	445.1399	18617	15.7	14552	3.5	0.0239

Meas. m/z	#	Ion Formula	m/z	err [ppm]	mSigma		Score	rdb	e⁻ Conf	N-Rule
443.1346	1	C25H19F3N2NaO	443.1342	-0.9	17.4	10	100.00	15.5	even	ok

Figure S7. ESI-HRMS spectrum of compound **G2**.

Synthesis of N-(4-acetylbenzyl)-4-phenyl-2-(trifluoromethyl)quinolin-7-aminium chloride (GA-1)

Under nitrogen atmosphere, a solution of **G2** (0.42 g, 1.0 mmol) in THF (20 mL) was cooled to 0 °C. The solution of HCl (1 M in THF) was then added dropwise until the reaction was complete. The resulting solid was collected by suction filtration and dried to afford **GA-1** as red solid (300 mg, 66% yield). ^{1}H NMR (600 MHz, DMSO- d_6) δ 8.19 (dd, J = 6.5, 3.0 Hz, 2H), 7.99 (s, 1H), 7.96 (d, J = 8.3 Hz, 2H), 7.88 (d, J = 8.1 Hz, 1H), 7.57 (dd, J = 8.7, 5.1 Hz, 5H), 7.41 (dd, J = 9.3, 2.0 Hz, 1H), 7.05 (s, 1H), 4.60 (s, 2H), 2.55 (s, 3H). ^{13}C NMR (151 MHz, DMSO- d_6) δ 197.92, 155.37, 151.53, 144.90, 136.13, 131.19, 129.81, 129.41, 129.24, 129.00, 128.37, 127.72, 127.62, 124.56, 122.04, 114.81, 111.63, 46.15, 27.17. HR-MS (ESI) m/z for $C_{25}H_{20}F_3N_2O$ [M]$^+$: calcd. 421.1522, found: 421.1534.

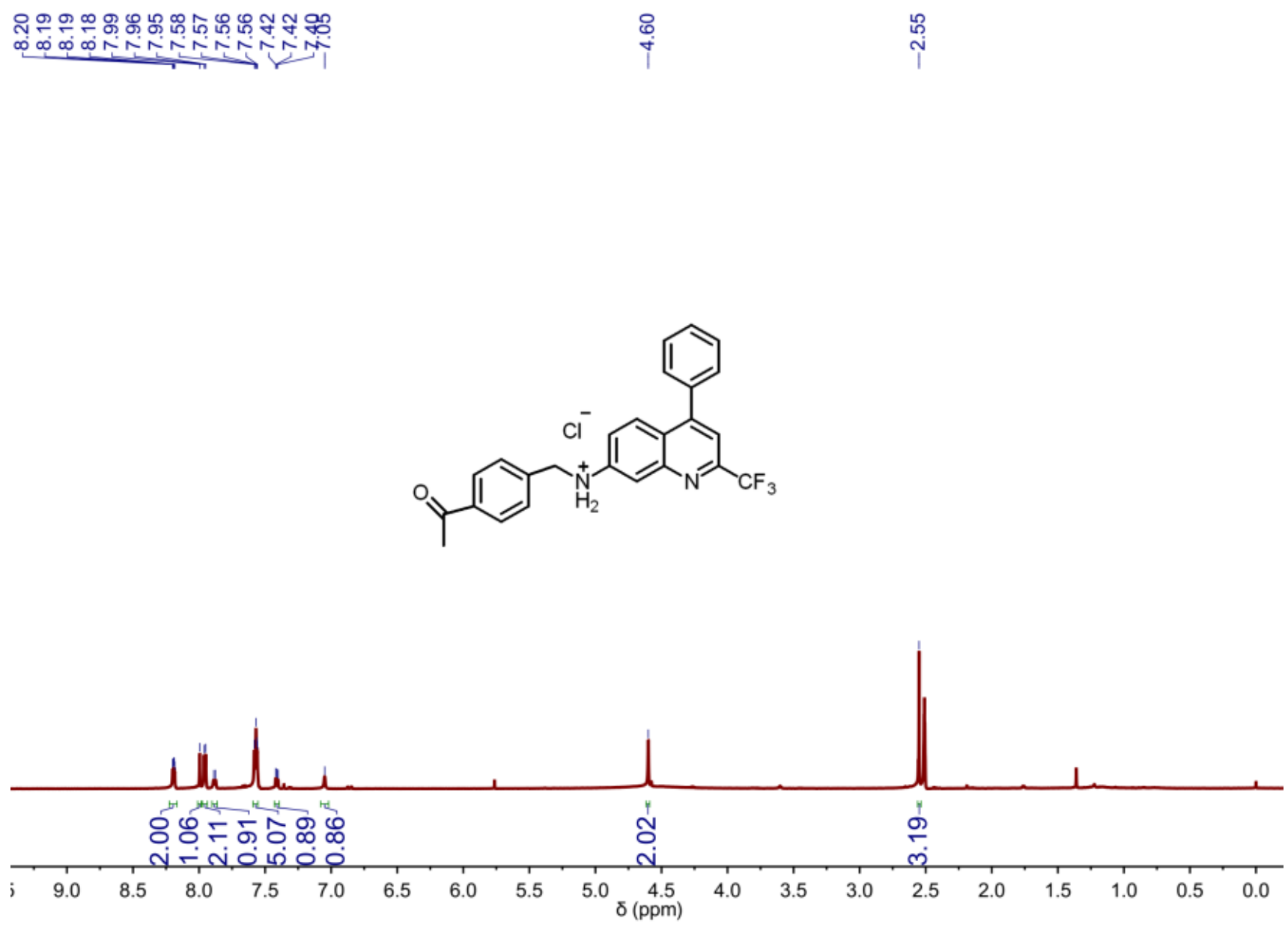

Figure S8. ^{1}H NMR spectrum of **GA-1** in DMSO- d_6 (25 °C).

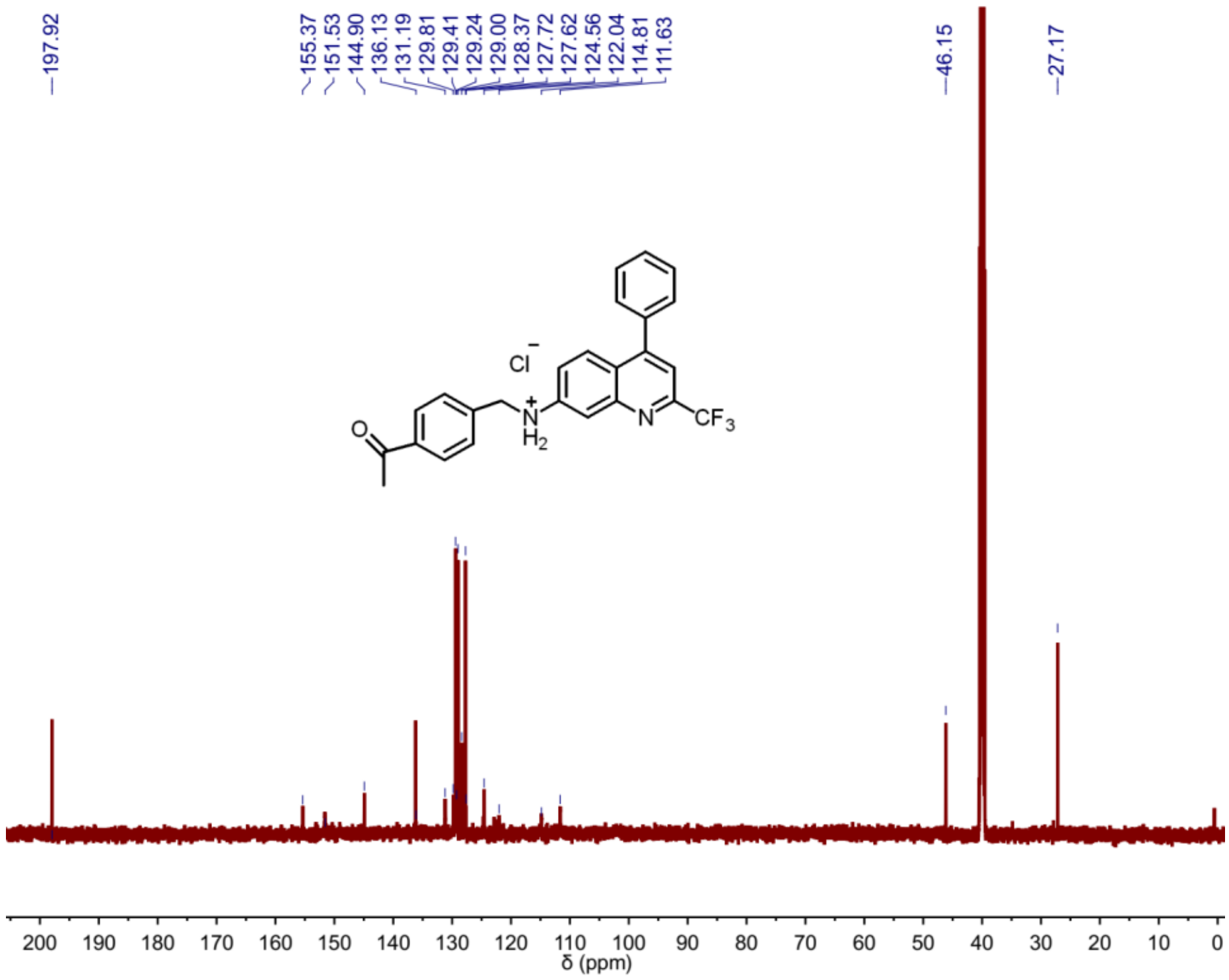

Figure S9. ^{13}C NMR spectrum of compound **GA-1** in DMSO- d_6 (25 °C).

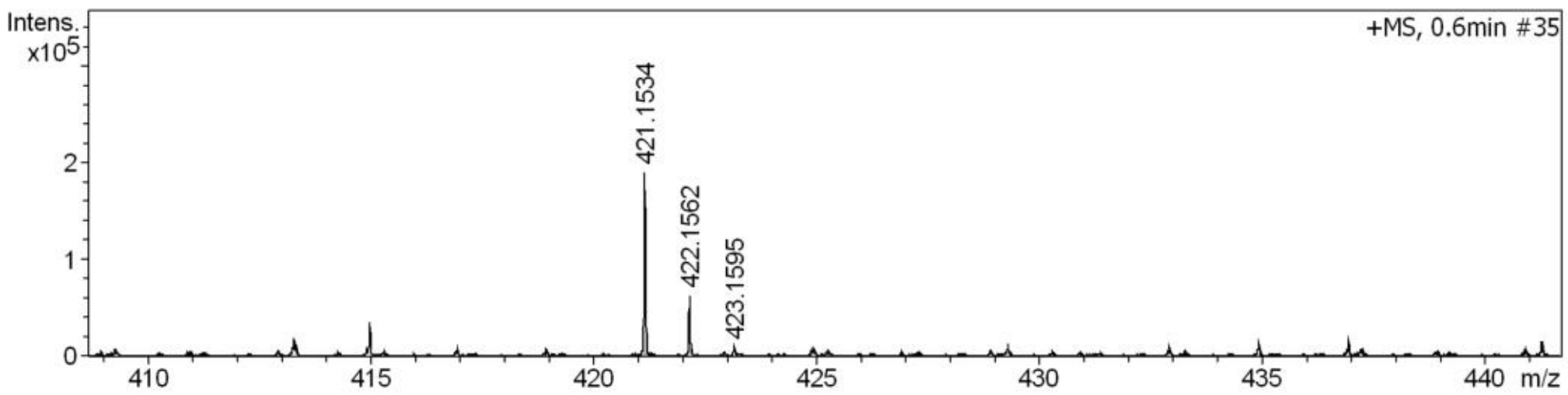

#	m/z	Res.	S/N	I	I %	FWHM
1	421.1534	15291	152.5	188956	100.0	0.0275
2	422.1562	17163	50.3	62268	33.0	0.0246
3	423.1595	15456	7.2	8904	4.7	0.0274

Meas. m/z	#	Ion Formula	m/z	err [ppm]	mSigma	#	Score	rdb	e⁻ Conf	N-Rule
421.1534	1	C25H20F3N2O	421.1522	-2.7	25.2	1	100.00	15.5	even	ok

Figure S10. ESI-HRMS spectrum of compound **GA-1**.

Synthesis of N-(4-phenyl-2-(trifluoromethyl)quinolin-7-yl)isonicotinamide (G3)

4-pyridinecarboxylic acid (123 mg, 1.0 mmol) and HATU (292 mg, 1.0 mmol) were dissolved in DMF (10 mL) under nitrogen atmosphere. DIPEA (0.314 mL, 1.0 mmol) was added via syringe, and the mixture was stirred at room temperature for 5 min. Then a solution of **G1** (288 mg, 1.0 mmol) and DIPEA (0.314 mL, 1.0 mmol) in DMF (10 mL) was added, and the reaction mixture was stirred for 12 h. The DMF was removed under reduced pressure, and the residue was partitioned between dichloromethane and water. The organic phase was dried over anhydrous Na_2SO_4 and

concentrated to give the crude product **G3**, which was used directly in the next step without further purification.

Synthesis of 1-methyl-4-((4-phenyl-2-(trifluoromethyl)quinolin-7-yl)carbamoyl)pyridin-1-ium (GA-2)

The crude **G3** from the previous step was dissolved in acetonitrile (10 mL) and heated until completely dissolved. Iodomethane (1 mL) was then added, and the mixture was heated at reflux until the reaction was complete. Then ethyl acetate was added until a large amount of solid precipitated. The solid was collected by suction filtration and subjected to anion exchange using chloride ion exchange resins, followed by drying to afford **GA-2** as yellow solid (164 mg, 37% yield). ^{1}H NMR (600 MHz, DMSO-d_6) δ 11.42 (s, 1H), 9.28 (d, J = 6.3 Hz, 2H), 8.89 (s, 1H), 8.63 (d, J = 6.3 Hz, 2H), 8.43-8.33 (m, 3H), 8.19 (d, J = 8.4 Hz, 1H), 8.09 (d, J = 10.7 Hz, 1H), 7.64-7.55 (m, 3H), 4.49 (s, 3H). ^{13}C NMR (151 MHz, DMSO-d_6) δ 161.73, 156.50, 148.92, 147.83, 146.47, 139.95, 137.24, 130.38, 128.95, 127.42, 125.91, 124.10, 122.57, 118.70, 117.97, 115.19, 48.22. HR-MS (ESI) m/z for $C_{23}H_{17}F_3N_3O$ $[M]^{+}$: calcd. 408.1318, found: 408.1328.

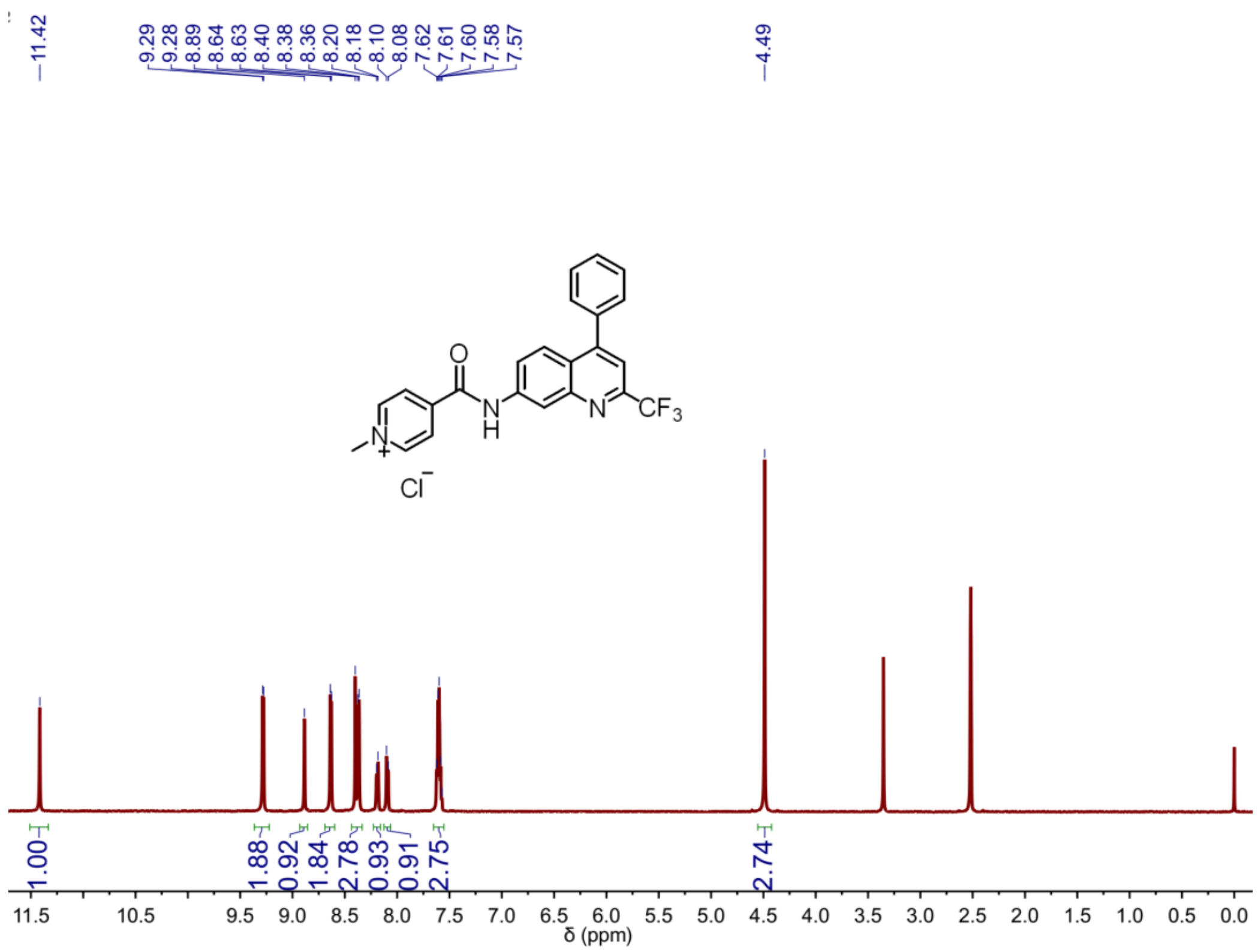

Figure S11. ^{1}H NMR spectrum of **GA-2** in DMSO-d_6 (25 °C).

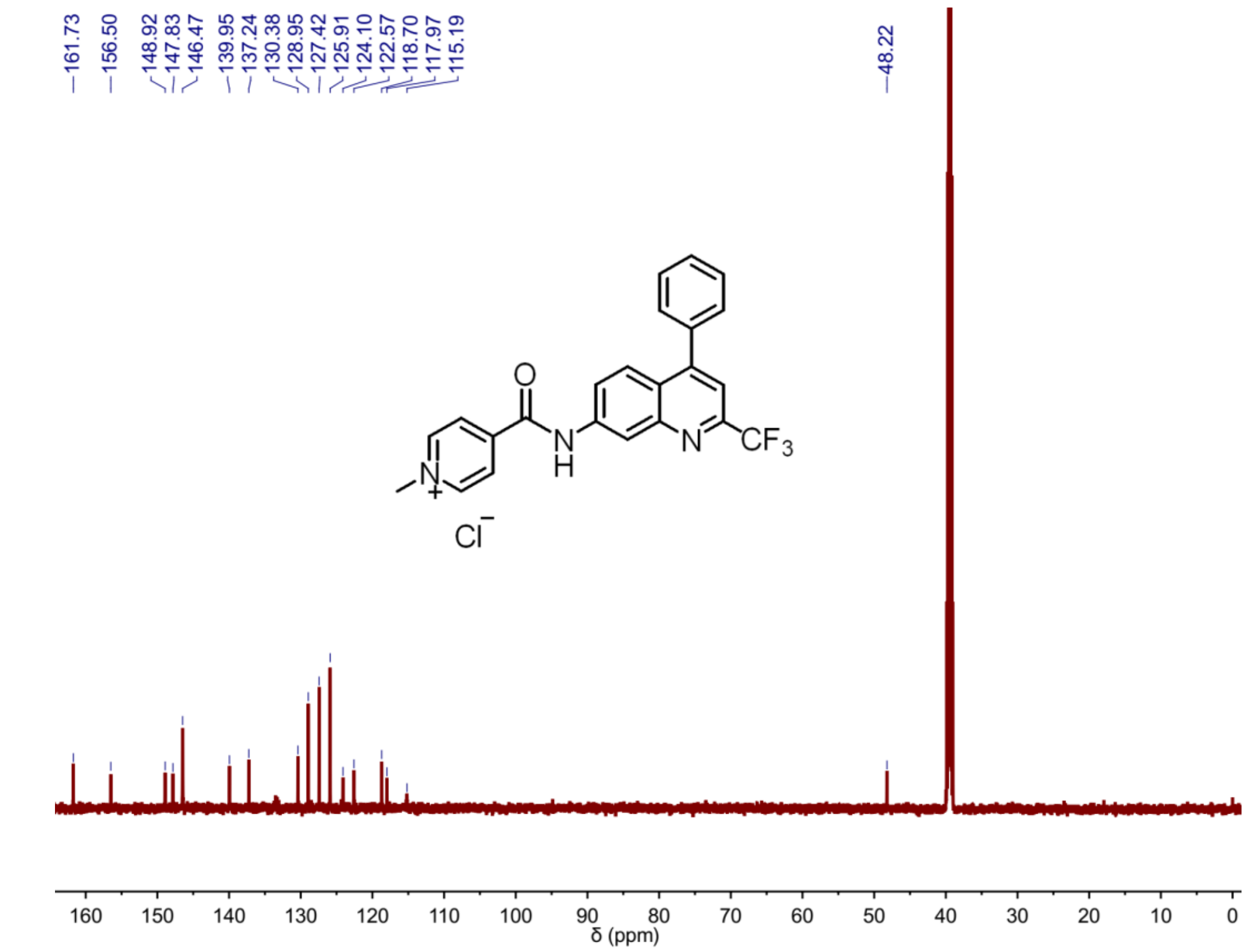

Figure S12. ^{13}C NMR spectrum of compound **GA-2** in DMSO-d_6 (25 °C).

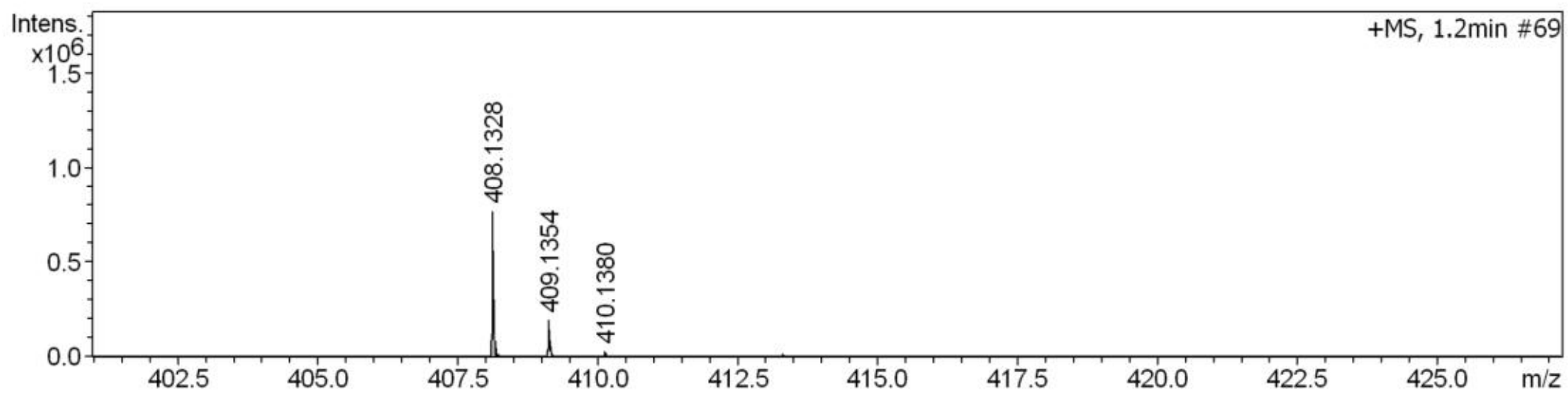

#	m/z	Res.	S/N	I	I %	FWHM
1	408.1328	22145	844.6	767644	100.0	0.0184
2	409.1354	20613	212.4	192764	25.1	0.0198
3	410.1380	18432	25.5	23140	3.0	0.0223

Meas. m/z	#	Ion Formula	m/z	err [ppm]	mSigma	#	Score	rdb	e⁻ Conf	N-Rule
408.1328	1	C23H17F3N3O	408.1318	-2.5	6.9	1	70.52	15.5	even	ok

Figure S13. ESI-HRMS spectrum of compound **GA-2**.

Synthesis of 4-phenyl-N-(pyridin-4-ylmethyl)-2-(trifluoromethyl)quinolin-7-amine (G4)

G1 (126 mg, 0.44 mmol) and 4-pyridinecarboxaldehyde (47 mg, 0.44 mmol) were dissolved in methanol (5 mL) under nitrogen atmosphere. One drop of glacial acetic acid was added, and the mixture was heated at reflux for 4 h. After confirming complete consumption of the starting materials, the reaction mixture was cooled to 0 °C, and sodium borohydride (50 mg, 1.32 mmol)

was added. The mixture was then allowed to warm to room temperature and stirred for 12 h. The reaction was quenched with saturated aqueous sodium bicarbonate, and the resulting mixture was extracted with dichloromethane. The combined organic phases were dried over anhydrous Na_2SO_4 and concentrated under reduced pressure. The crude product was purified by silica gel column chromatography using dichloromethane/ethyl acetate (1/1, v/v) as eluent to afford compound **G4** as yellow solid (100 mg, 60% yield). ^{1}H NMR (600 MHz, DMSO-d_6) δ 8.53 (d, J = 5.9 Hz, 2H), 8.21 (d, J = 7.6 Hz, 2H), 7.98 (s, 1H), 7.83 (d, J = 9.1 Hz, 1H), 7.56-7.50 (m, 3H), 7.42 (d, J = 4.9 Hz, 2H), 7.33 (dd, J = 9.2, 2.3 Hz, 1H), 6.91 (d, J = 2.1 Hz, 1H), 4.56 (d, J = 5.9 Hz, 2H), 3.31 (s, 1H). ^{13}C NMR (151 MHz, DMSO-d_6) δ 155.58, 150.81, 149.98, 149.59, 148.45, 137.82, 129.79, 128.75, 127.08, 123.66, 122.10, 120.98, 120.60, 113.44, 104.48, 44.80. HR-MS (ESI) m/z for $C_{22}H_{17}F_3N_3$ $[M+H]^+$: calcd. 380.1369, found: 380.1376.

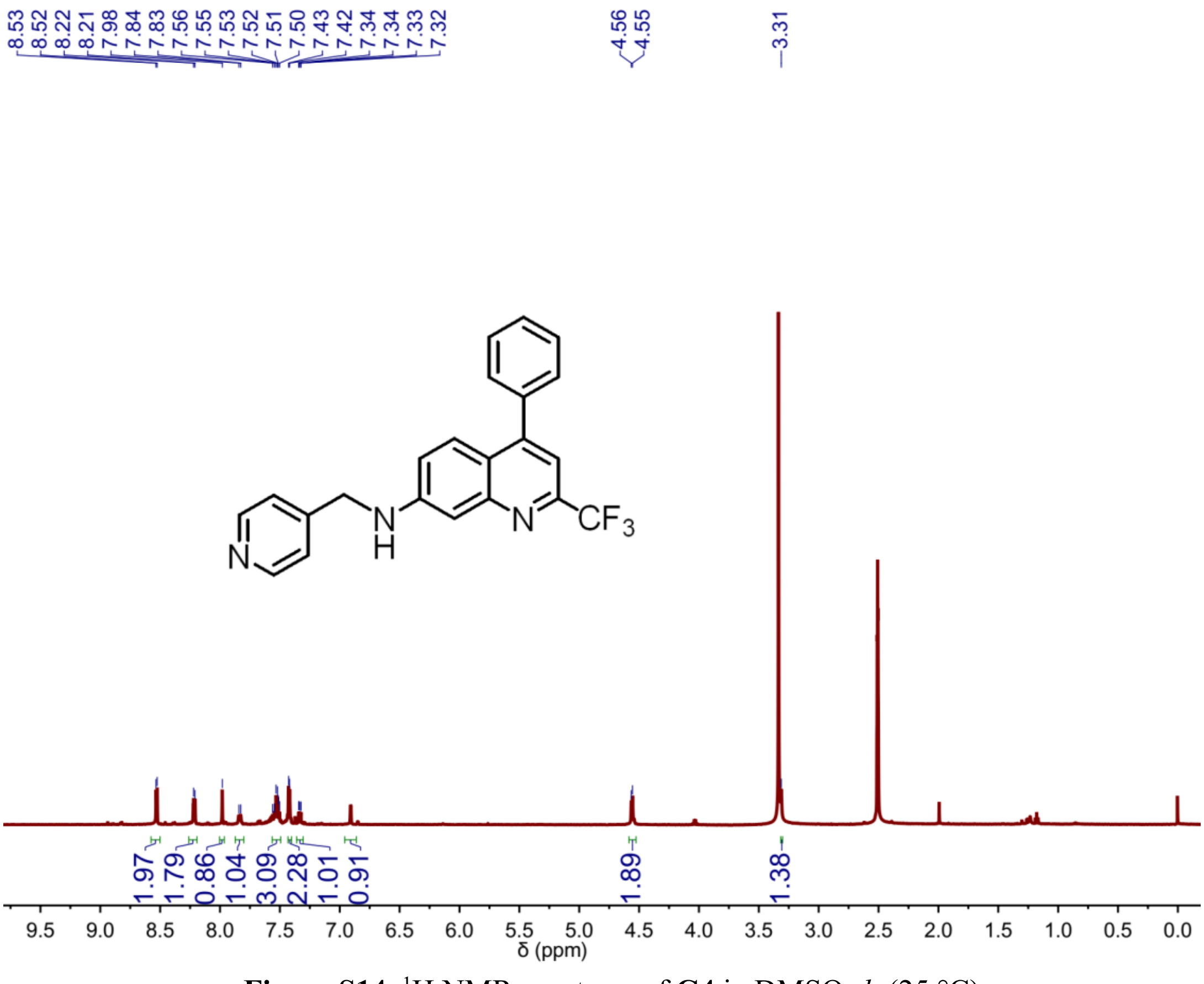

Figure S14. ^{1}H NMR spectrum of **G4** in DMSO-d_6 (25 °C).

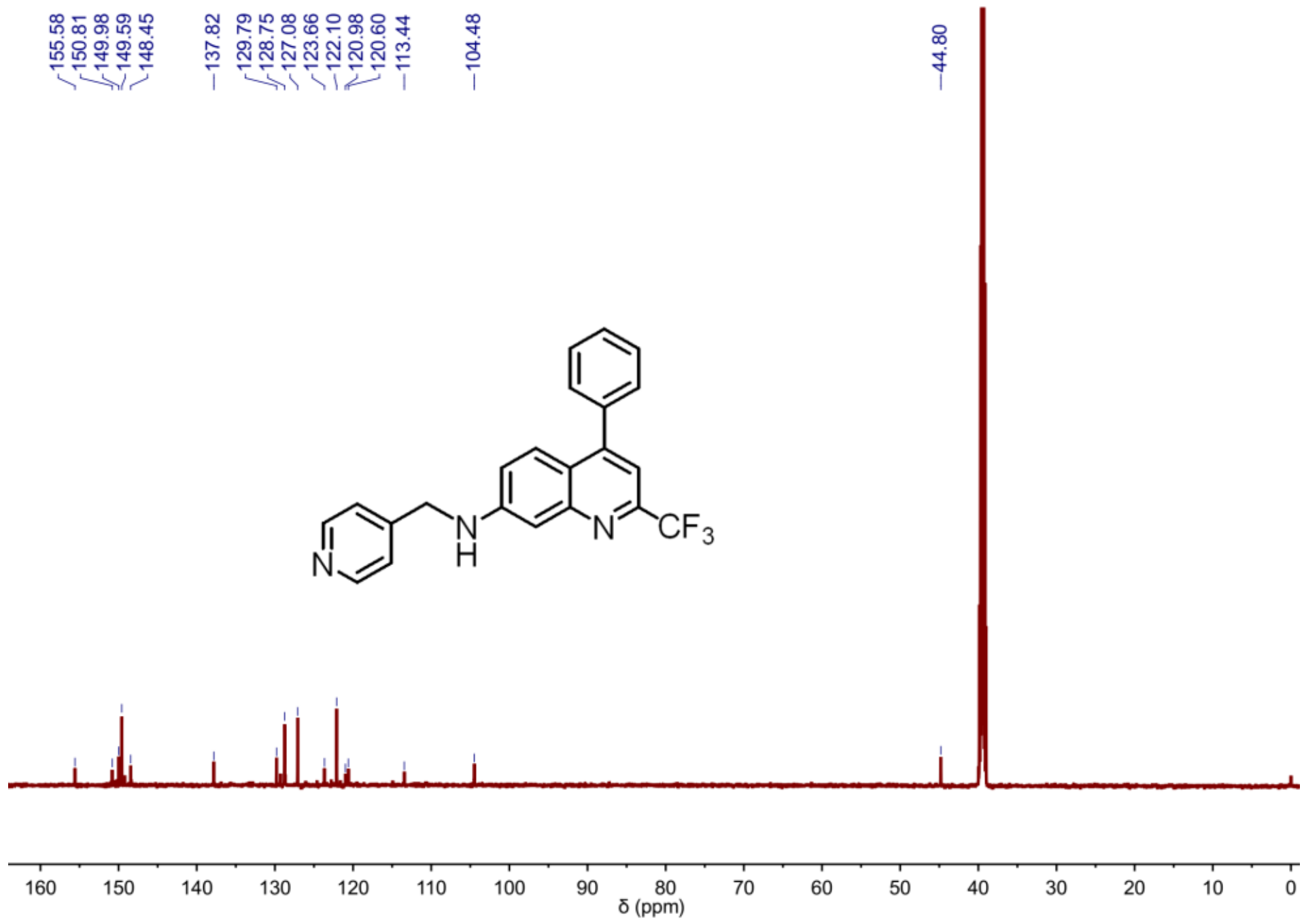

Figure S15. ^{13}C NMR spectrum of compound **G4** in DMSO-d_6 (25 °C).

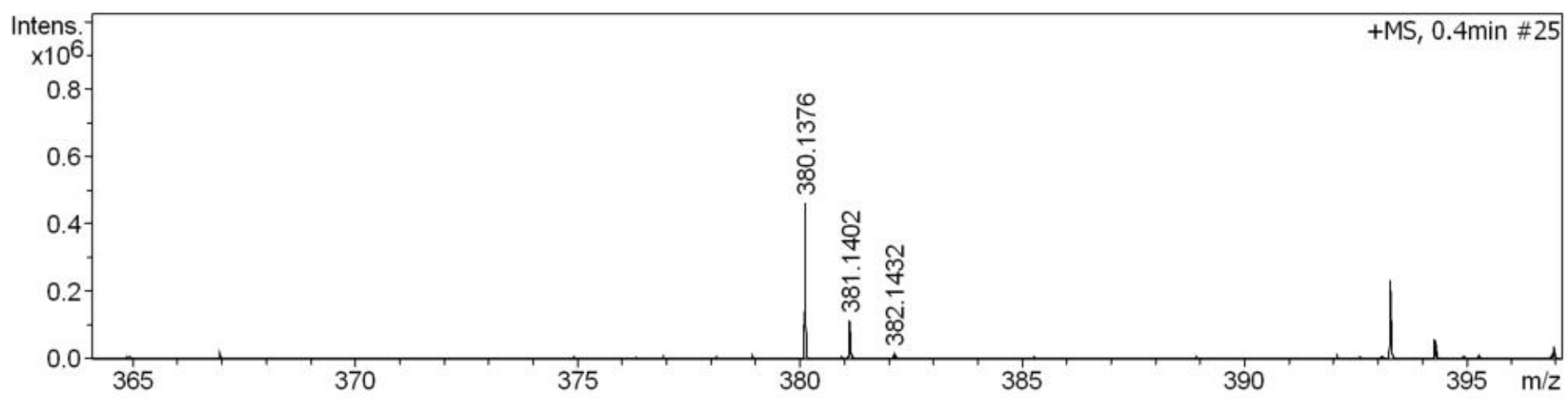

#	m/z	Res.	S/N	I	I %	FWHM
1	380.1376	19396	482.7	461968	100.0	0.0196
2	381.1402	17161	118.7	113592	24.6	0.0222
3	382.1432	14273	15.1	14436	3.1	0.0268

Meas. m/z	#	Ion Formula	m/z	err [ppm]	mSigma	#	Score	rdb	e⁻ Conf	N-Rule
380.1376	1	C22H17F3N3	380.1369	-1.7	2.9	1	100.00	14.5	even	ok

Figure S16. ESI-HRMS spectrum of compound **G4**.

Synthesis of 1-methyl-4-(((4-phenyl-2-(trifluoromethyl)quinolin-7-yl)amino)methyl)pyridin-1-ium (GA-3)

G4 (379 mg, 1.0 mmol) was dissolved in acetonitrile (10 mL) and heated with stirring until complete dissolution. Iodomethane (1 mL) was then added, and the mixture was heated at reflux for an additional 12 h. Then ethyl acetate was added until a large amount of solid precipitated. The solid was collected by suction filtration and subjected to anion exchange using chloride ion exchange resins, followed by drying to afford **GA-3** as yellow solid (313 mg, 73% yield). ^1H NMR (600 MHz, DMSO-d_6) δ 8.97 (d, J = 6.4 Hz, 2H), 8.47 (d, J = 6.6 Hz, 2H), 8.25 (d, J = 6.9 Hz, 2H), 8.07 (s,

1H), 7.83 (d, J = 7.8 Hz, 1H), 7.60-7.53 (m, 3H), 7.48 (d, J = 8.1 Hz, 1H), 7.29 (d, J = 11.0 Hz, 1H), 7.17 (d, J = 1.8 Hz, 1H), 4.22 (s, 3H), 2.51 (s, 2H, partially overlaps with the residual proton peak of DMSO). ^{13}C NMR (151 MHz, DMSO-d_6) δ 158.45, 155.97, 150.27, 147.68, 145.72, 145.48, 137.55, 130.08, 128.84, 127.14, 126.31, 124.14, 114.21, 111.90, 108.38, 58.05, 47.56. HR-MS (ESI) m/z for $C_{23}H_{19}F_3N_3$ [M]$^+$: calcd. 394.1526, found: 394.1531.

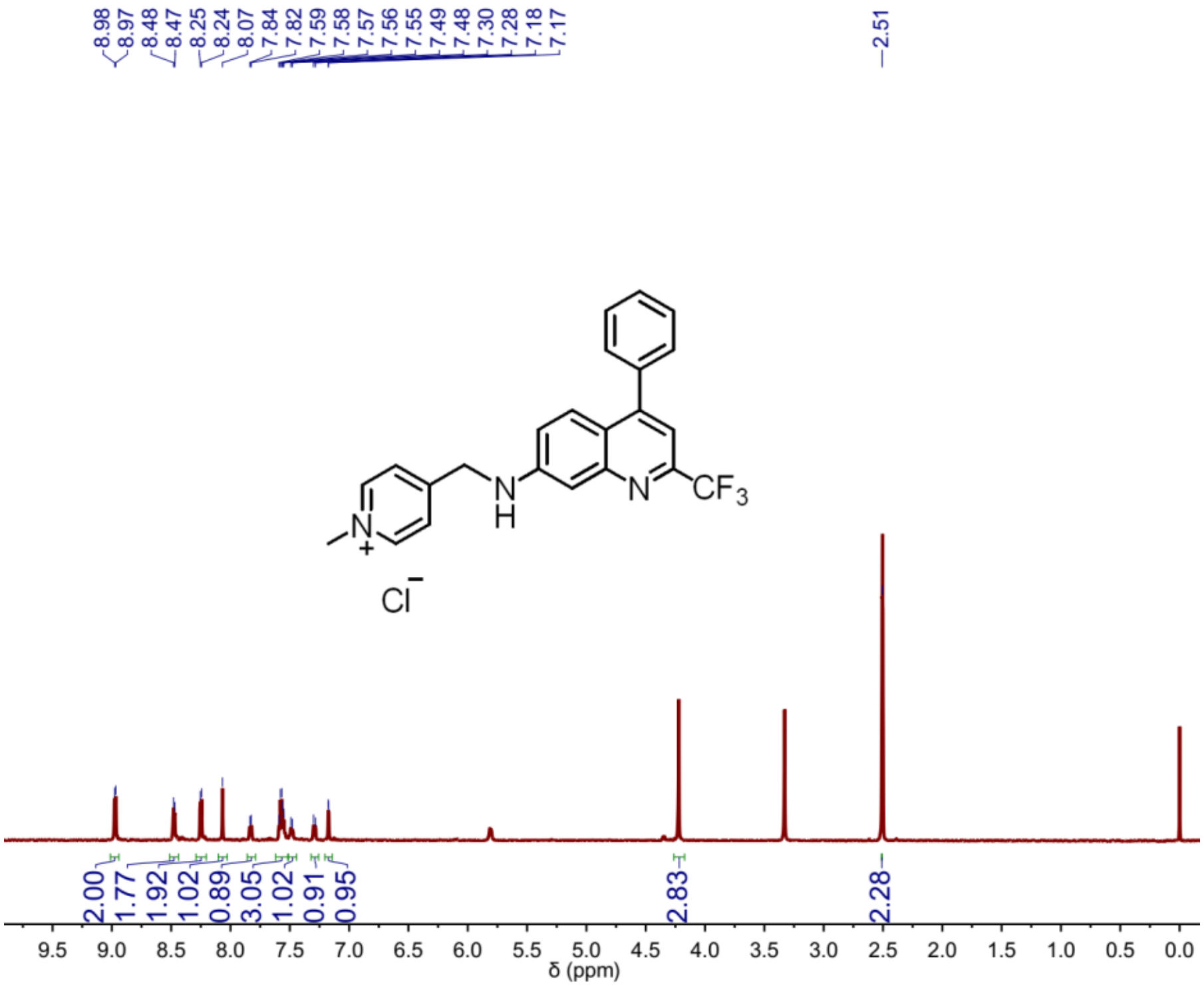

Figure S17. ^{1}H NMR spectrum of **GA-3** in DMSO-d_6 (25 °C).

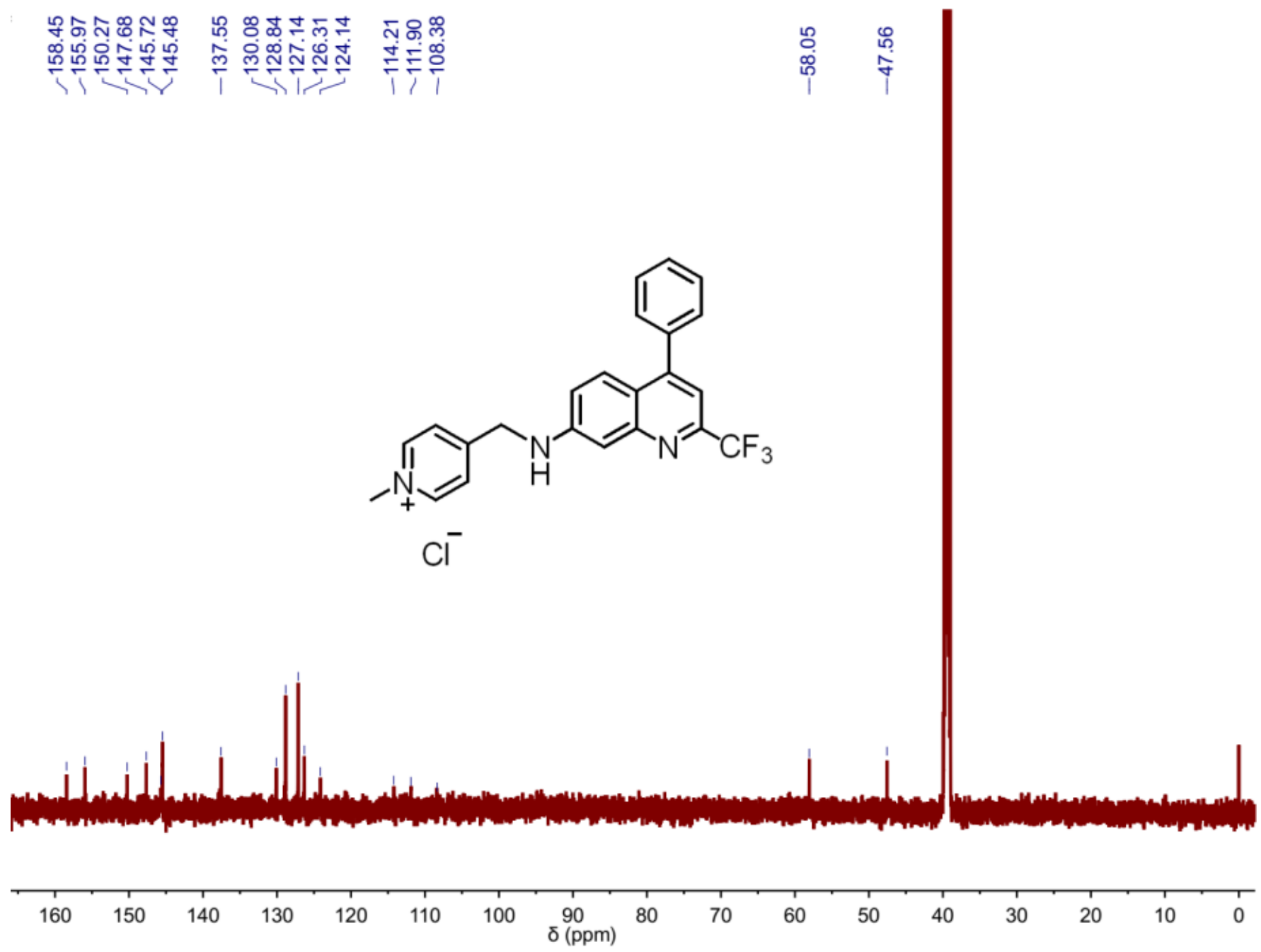

Figure S18. ^{13}C NMR spectrum of compound **GA-3** in DMSO-d_6 (25 °C).

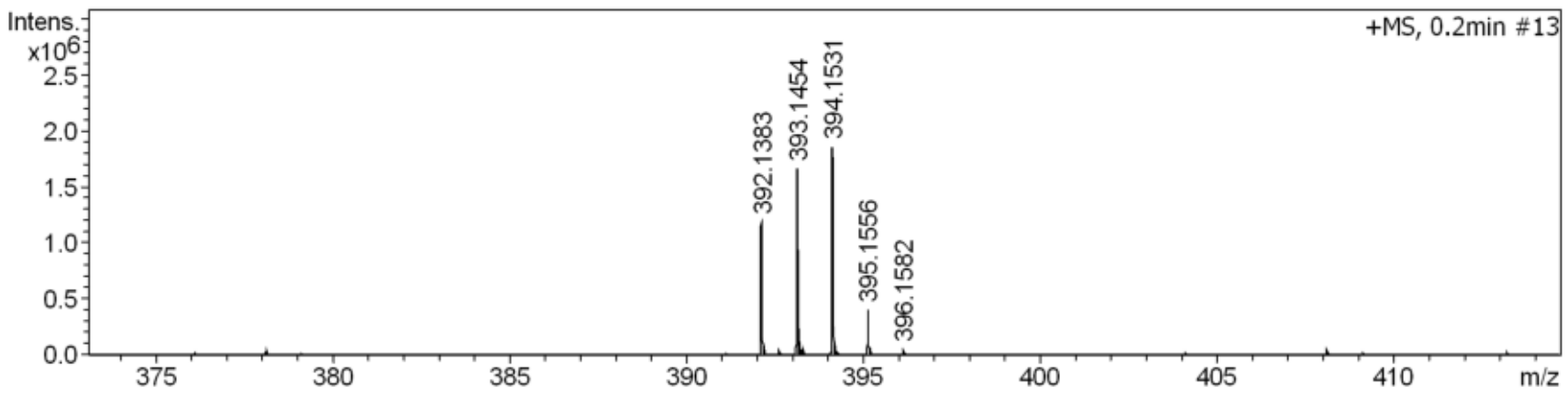

#	m/z	Res.	S/N	I	I %	FWHM
1	392.1383	21892	1267.2	1183728	63.9	0.0179
2	393.1454	21865	1776.8	1659620	89.6	0.0180
3	394.1531	21206	1984.1	1851584	100.0	0.0186
4	395.1556	19724	434.5	405068	21.9	0.0200
5	396.1582	16614	49.6	46188	2.5	0.0238

Meas. m/z	#	Ion Formula	m/z	err [ppm]	mSigma		Score	rdb	e¯ Conf	N-Rule
394.1531	1	C23H19F3N3	394.1526	-1.3	25.3	5	73.03	14.5	even	ok

Figure S19. ESI-HRMS spectrum of compound **GA-3**.

3. Optical measurements and biological experiments.

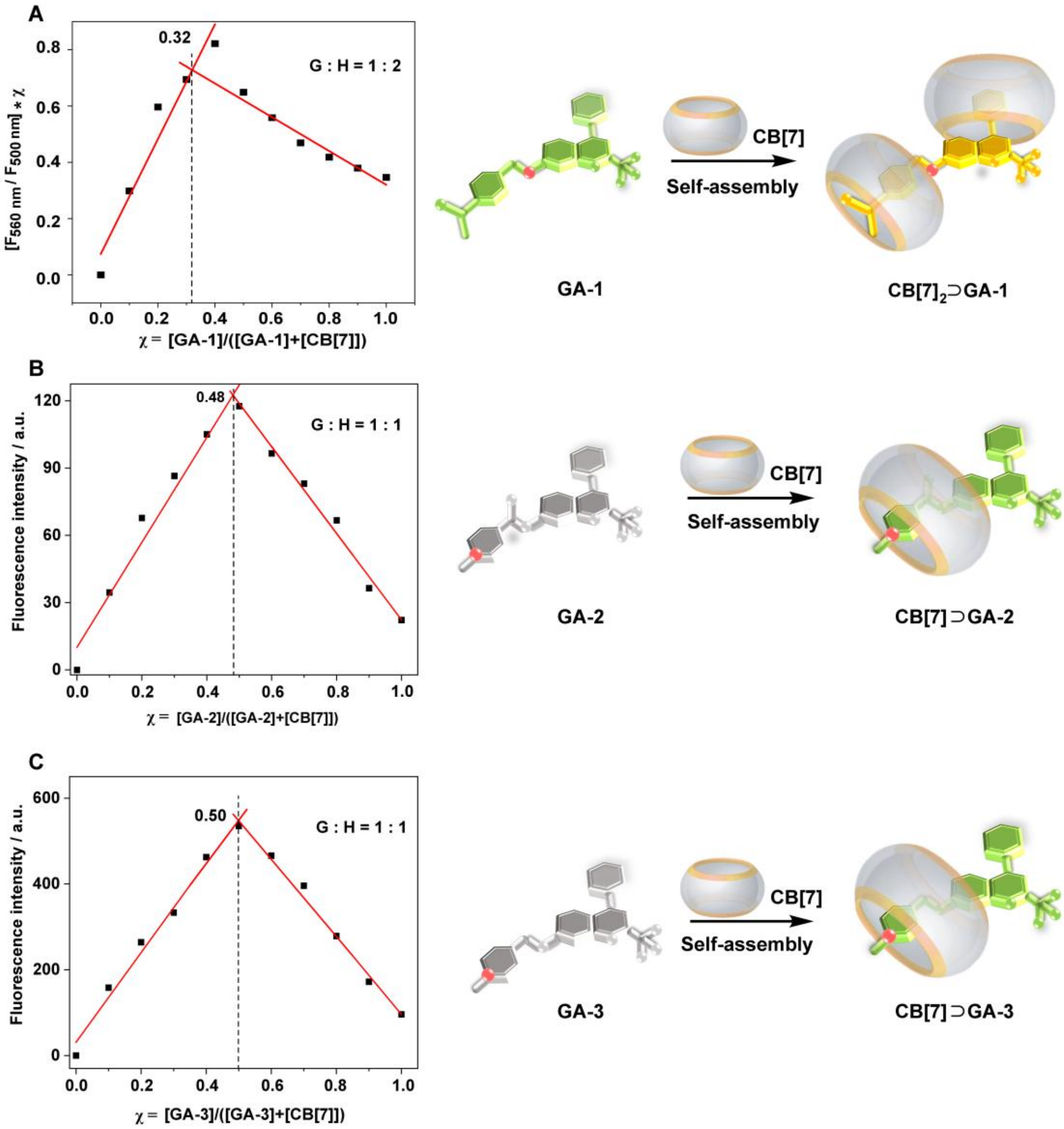

Figure S20. Job's plots and schematic illustrations of the self-assembly of different guest molecules with CB[7]: (A) **GA-1**; (B) **GA-2**; (C) **GA-3**. The anions (Cl^-) were omitted for clarity.

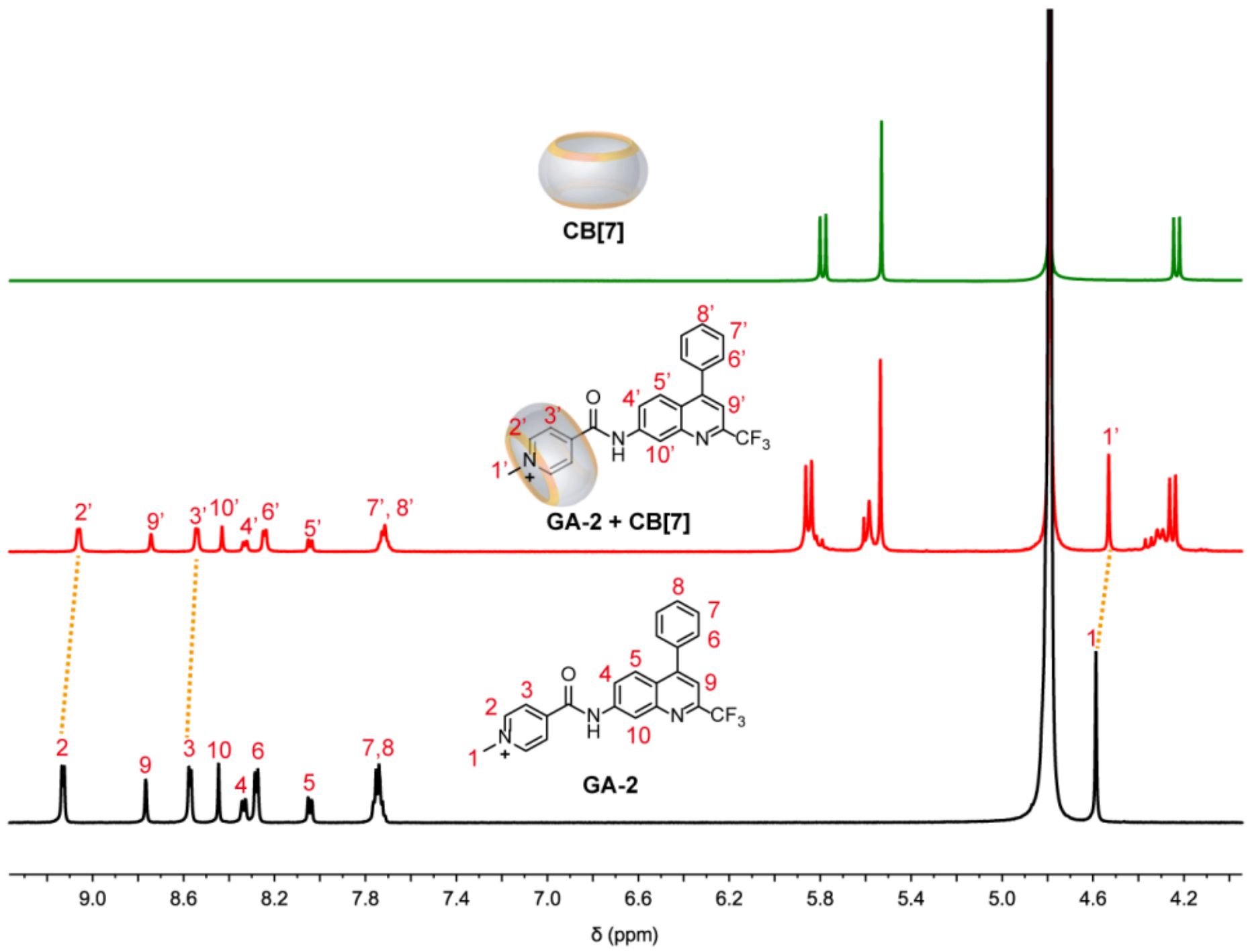

Figure S21. ^{1}H NMR spectra of **GA-2**, **GA-2** with CB[7], and CB[7] alone in D_2O. [CB[7]] = 1 mM, [**GA-2**] = 0.5 mM. The anions (Cl^-) were omitted for clarity.

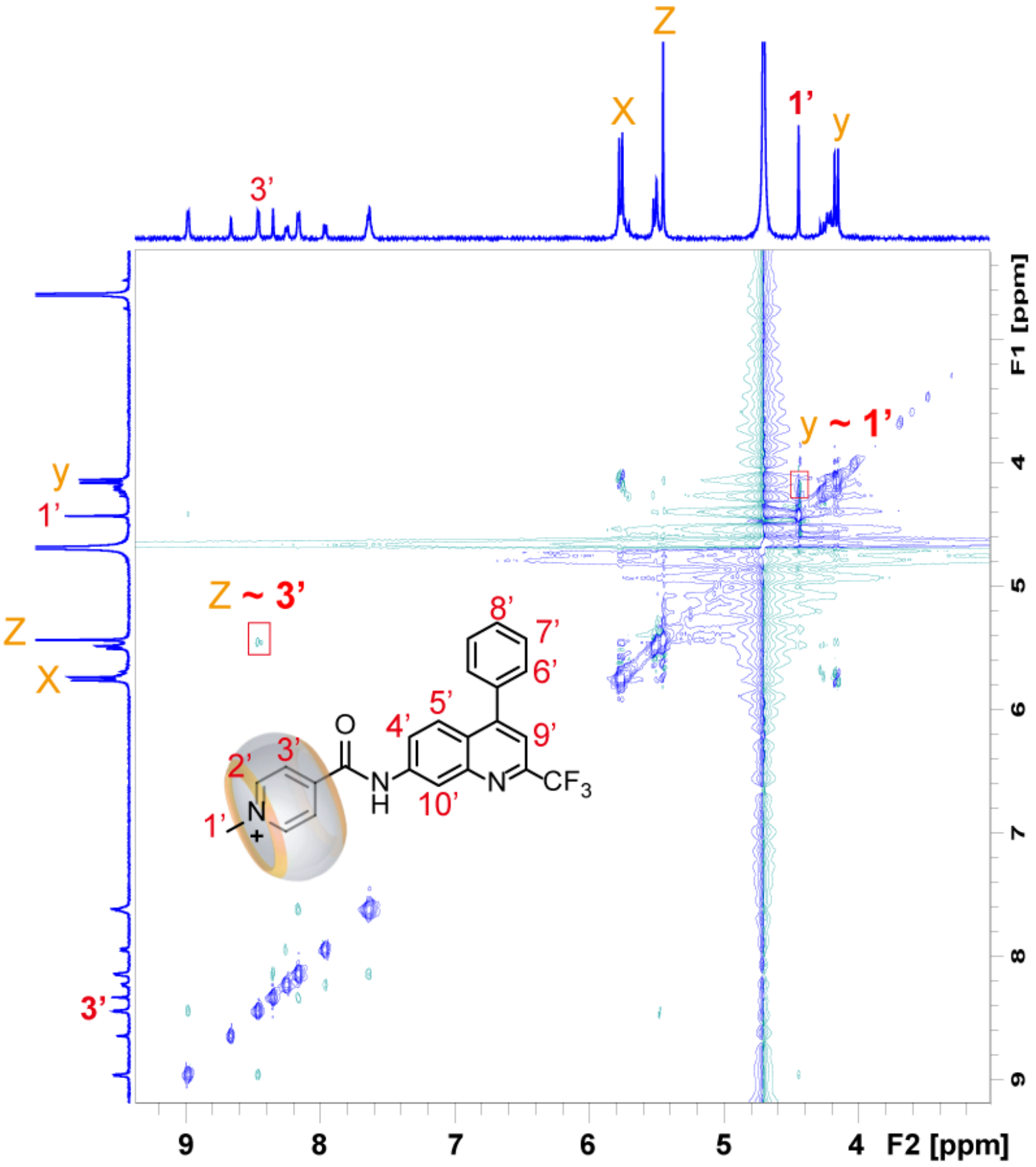

Figure S22. 2D ROESY NMR spectrum of the complex **CB[7]⊃GA-2**, in which [**GA-2**] = [CB[7]] = 1 mM in D_2O at 298 K. The anion (Cl^-) was omitted for clarity.

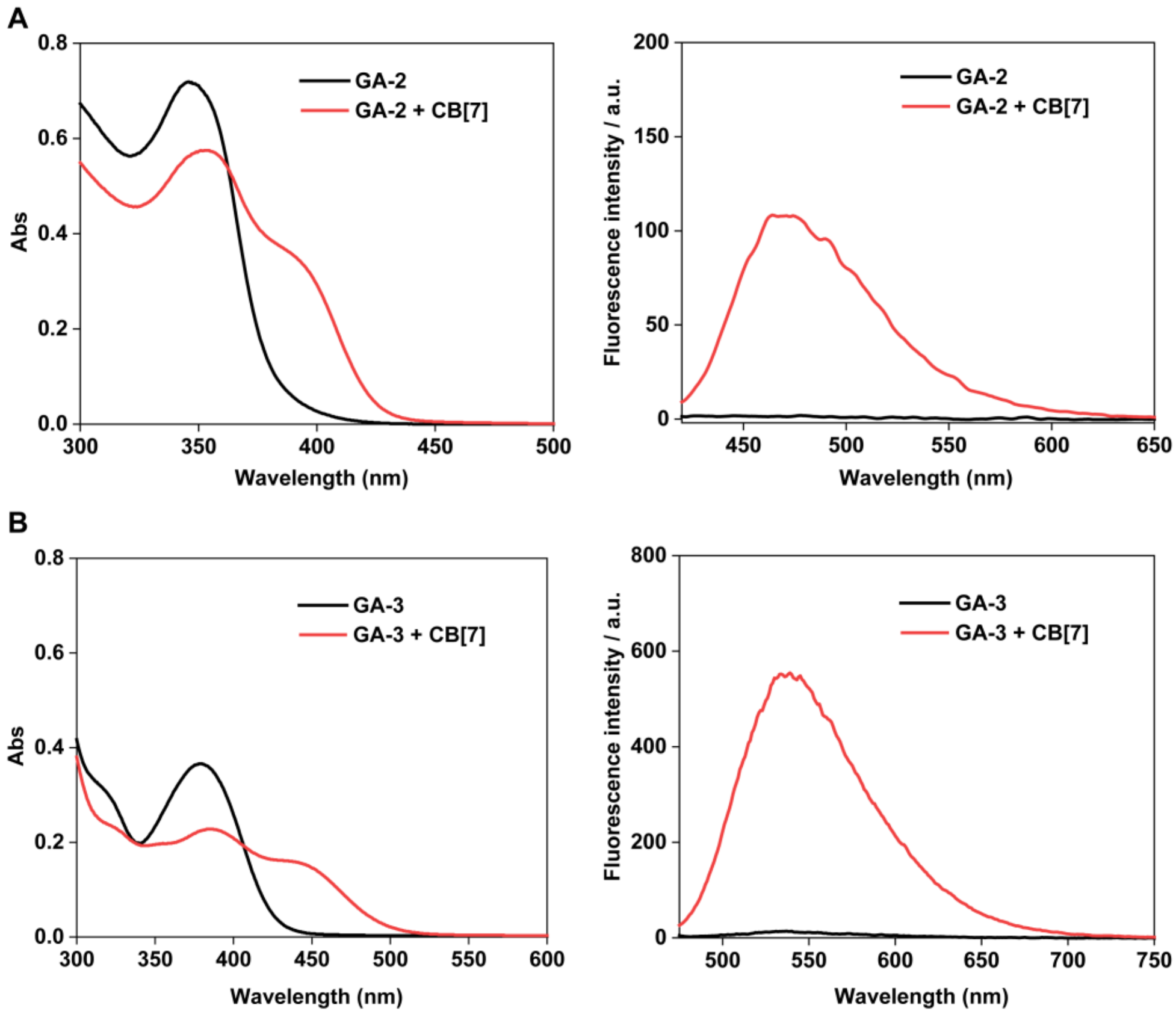

Figure S23. UV–Vis absorption and fluorescence emission spectra of different guest molecules upon self-assembly with CB[7]: (A) **GA-2** (λ_{ex} = 360 nm); (B) **GA-3** (λ_{ex} = 440 nm).

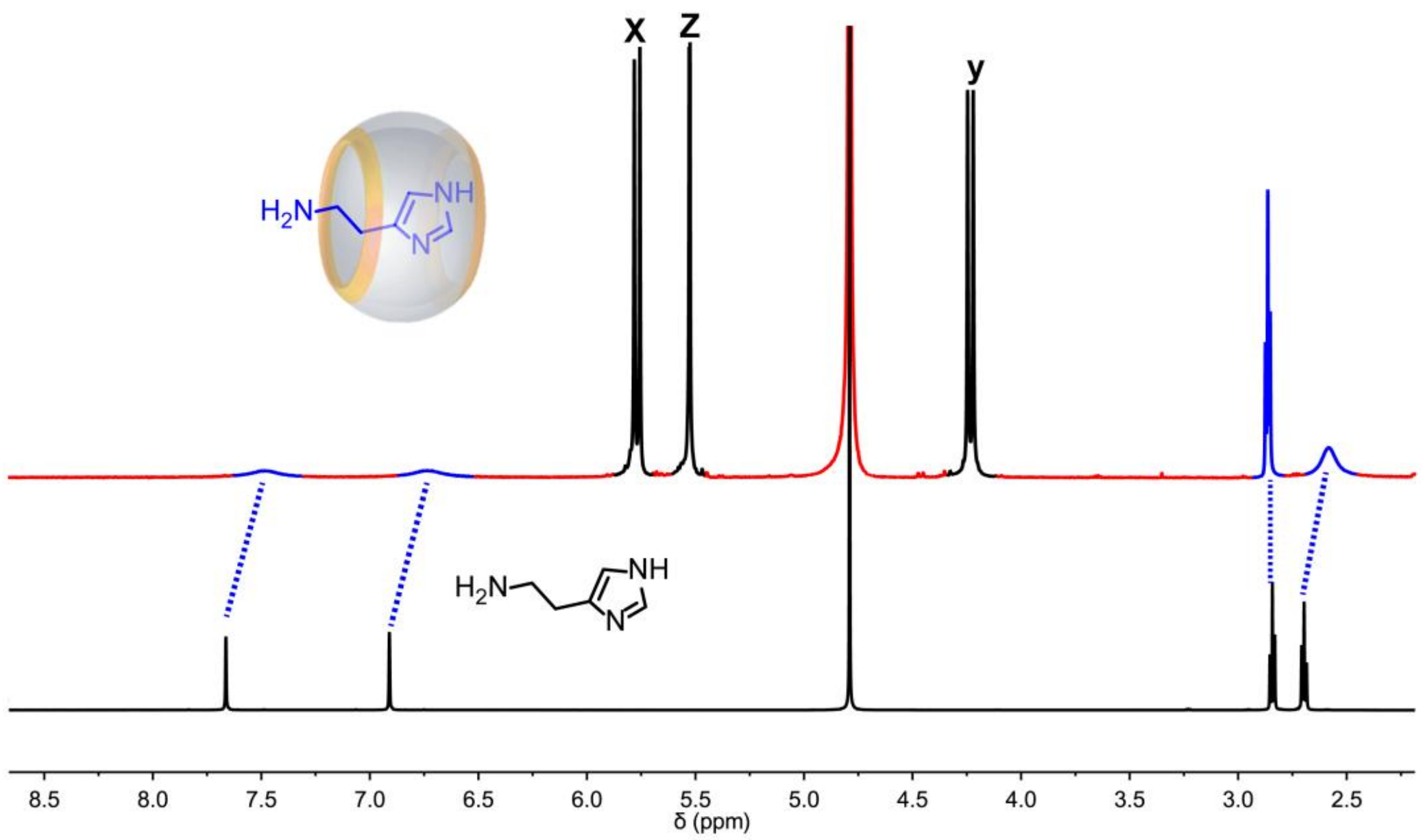

Figure S24. ^{1}H NMR spectra of HA and HA with CB[7] in D_2O. [CB[7]] = [HA] = 1 mM.

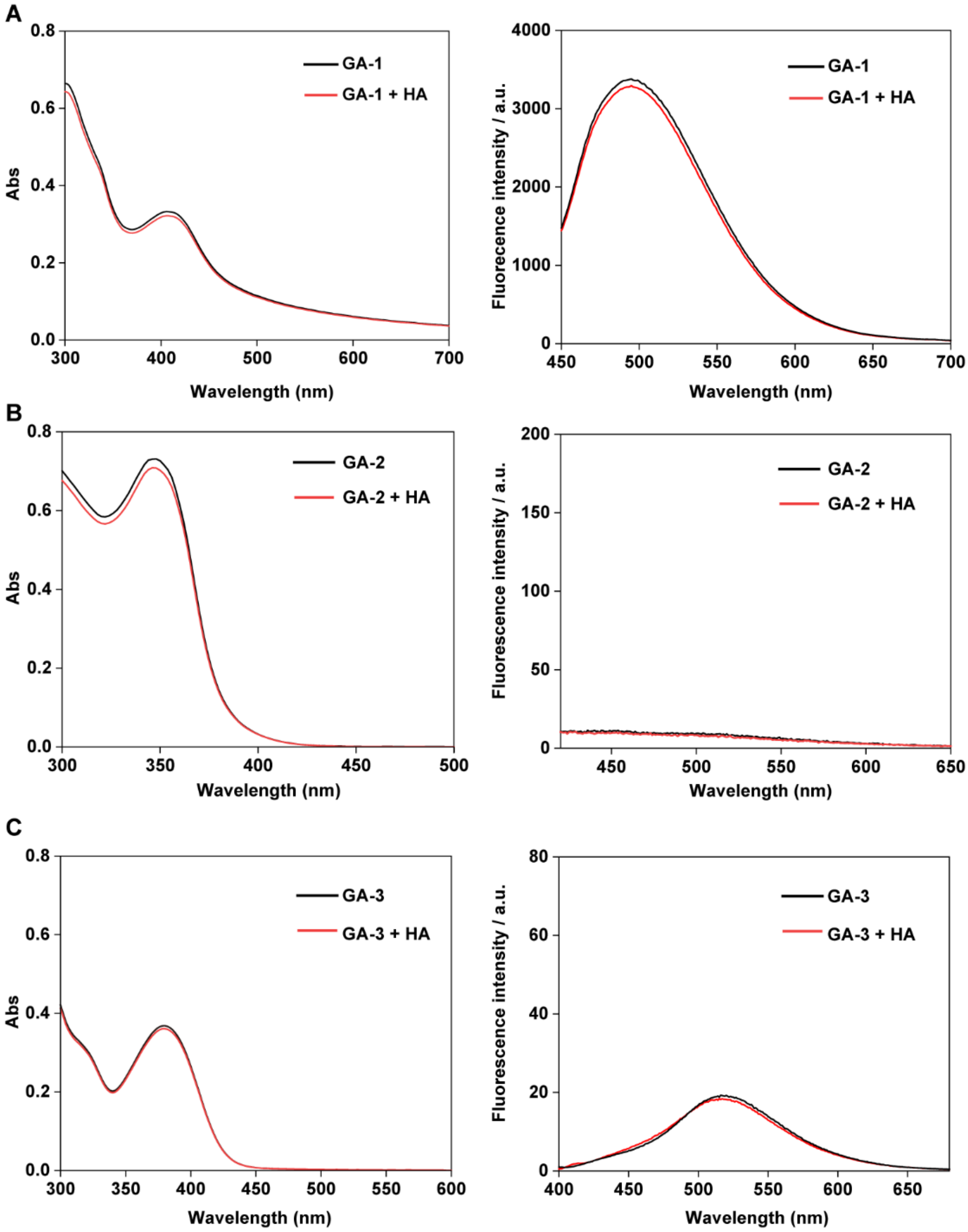

Figure S25. UV–Vis absorption and fluorescence emission spectra of guest dyes (30 μM) upon the addition of 2 equiv. of HA: (A) **GA-1** (λ_{ex} = 440 nm), (B) **GA-2** (λ_{ex} = 360 nm), and (C) **GA-3** (λ_{ex} = 440 nm) upon addition of 2 equiv. of HA.

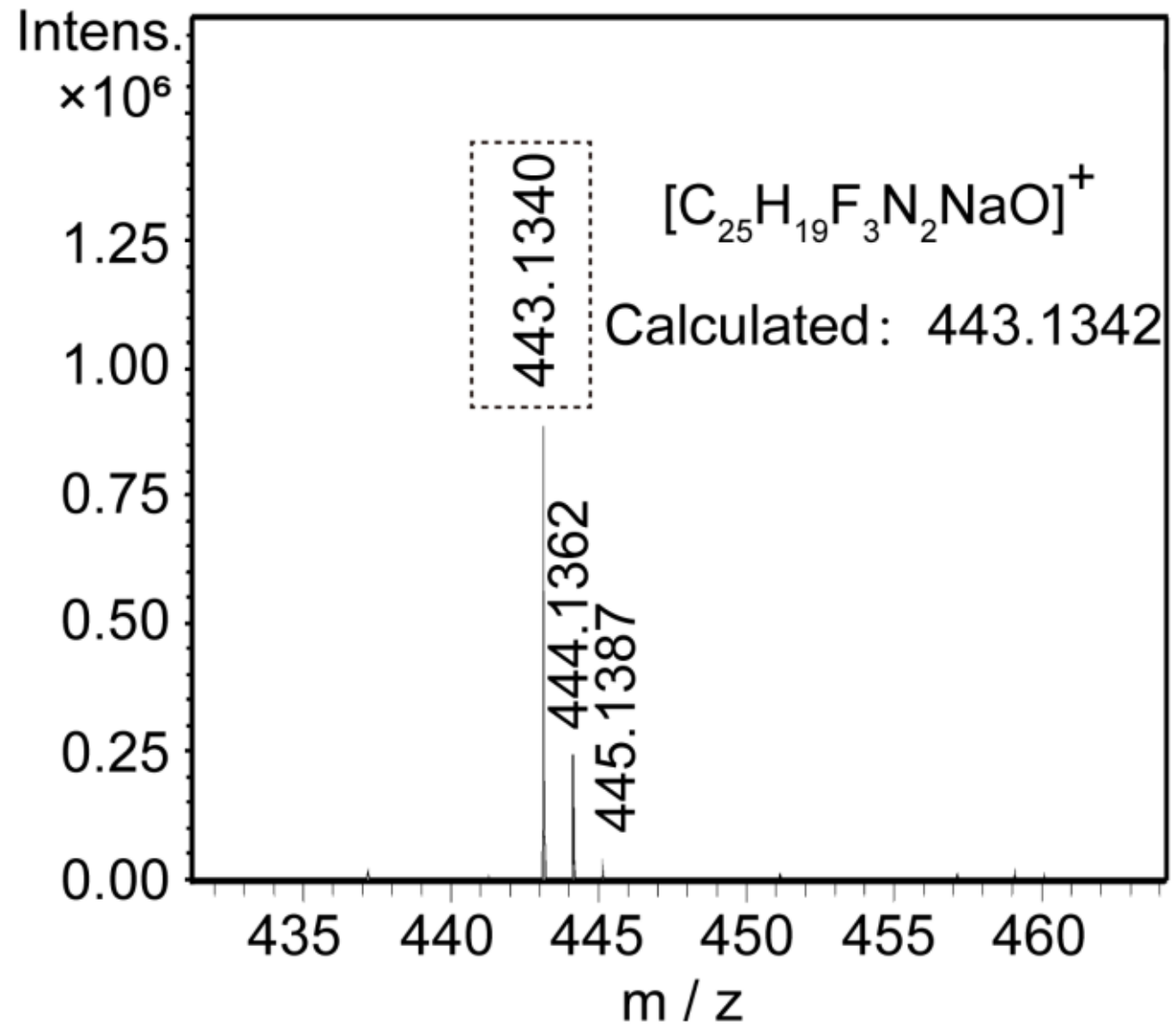

Figure S26. Mass spectrometry of the complex **CB[7]₂⊃GA-1** after addition of 2 equivalents of HA.

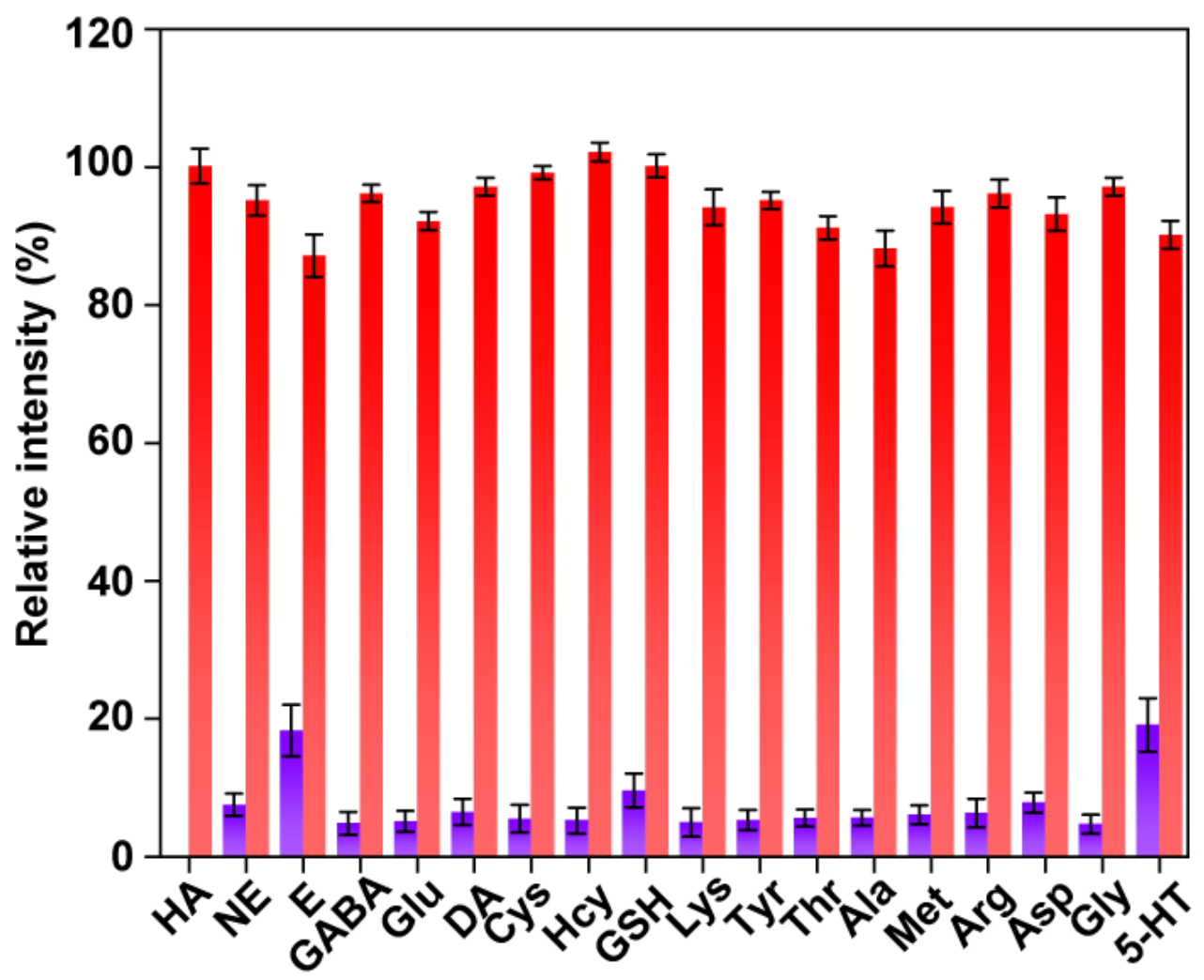

Figure S27. Competition tests of probe **CB[7]₂⊃GA-1** towards neurotransmitters, biothiols, and amino acids, including histamine (HA), norepinephrine (NE), epinephrine (E), γ-aminobutyric acid (GABA), glutamic acid (Glu), dopamine (DA), cysteine (Cys), homocysteine (Hcy), glutathione (GSH), lysine (Lys), tyrosine (Tyr), threonine (Thr), alanine (Ala), methionine (Met), arginine (Arg), aspartic acid (Asp), glycine (Gly), 5-hydroxytryptamine (5-HT). The concentration of GSH was 1mM, while other species are at 100 μM. Data are presented as mean ± S.D., n = 3 independent experiments, excited at 440 nm.

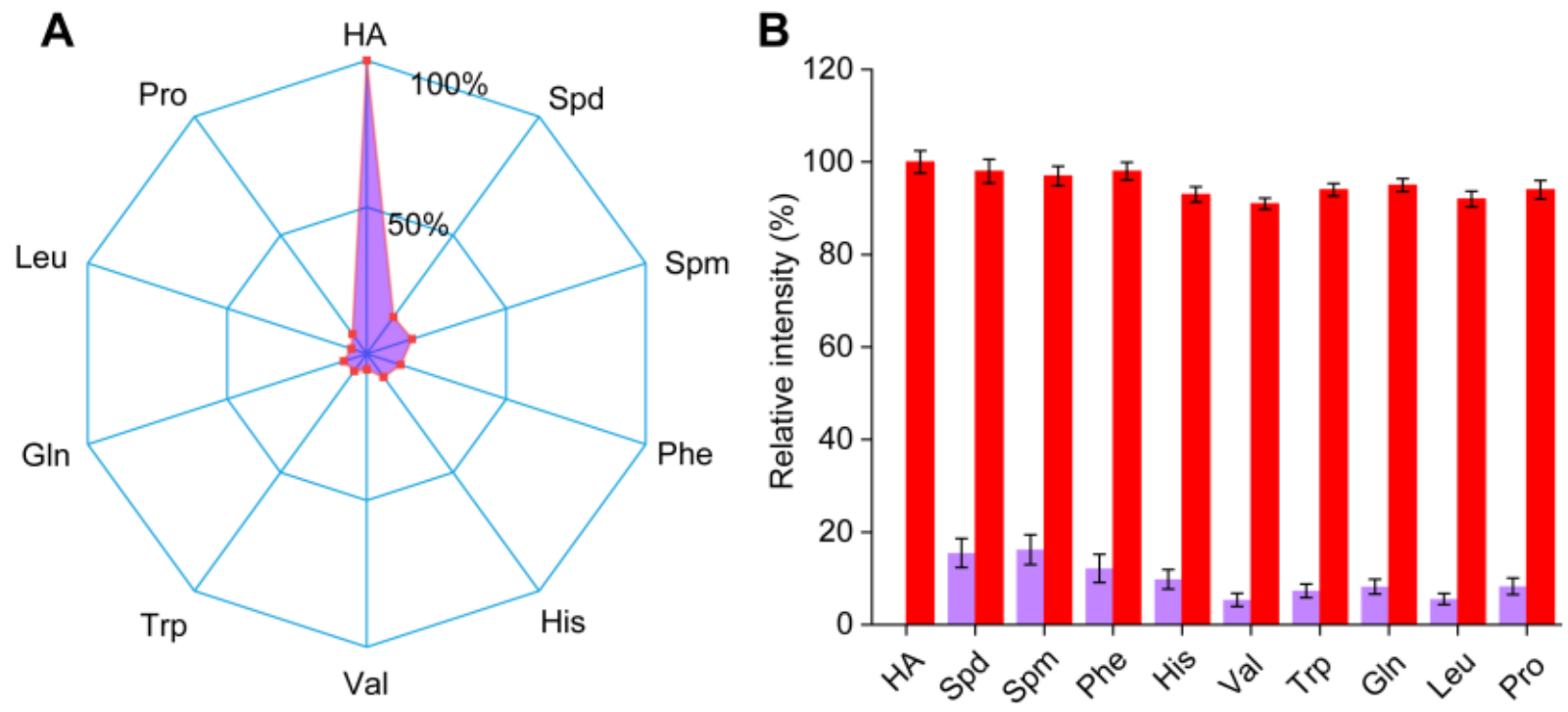

Figure S28. Selectivity (A) and competition (B) tests of probe **CB[7]$_2$⊃GA-1** in cell lysis buffer (10 mM, pH = 7.4, containing 1% DMSO) toward HA (100 μM) against representative amino acids (100 μM) and polyamines (10 μM), λ_{ex} = 440 nm.

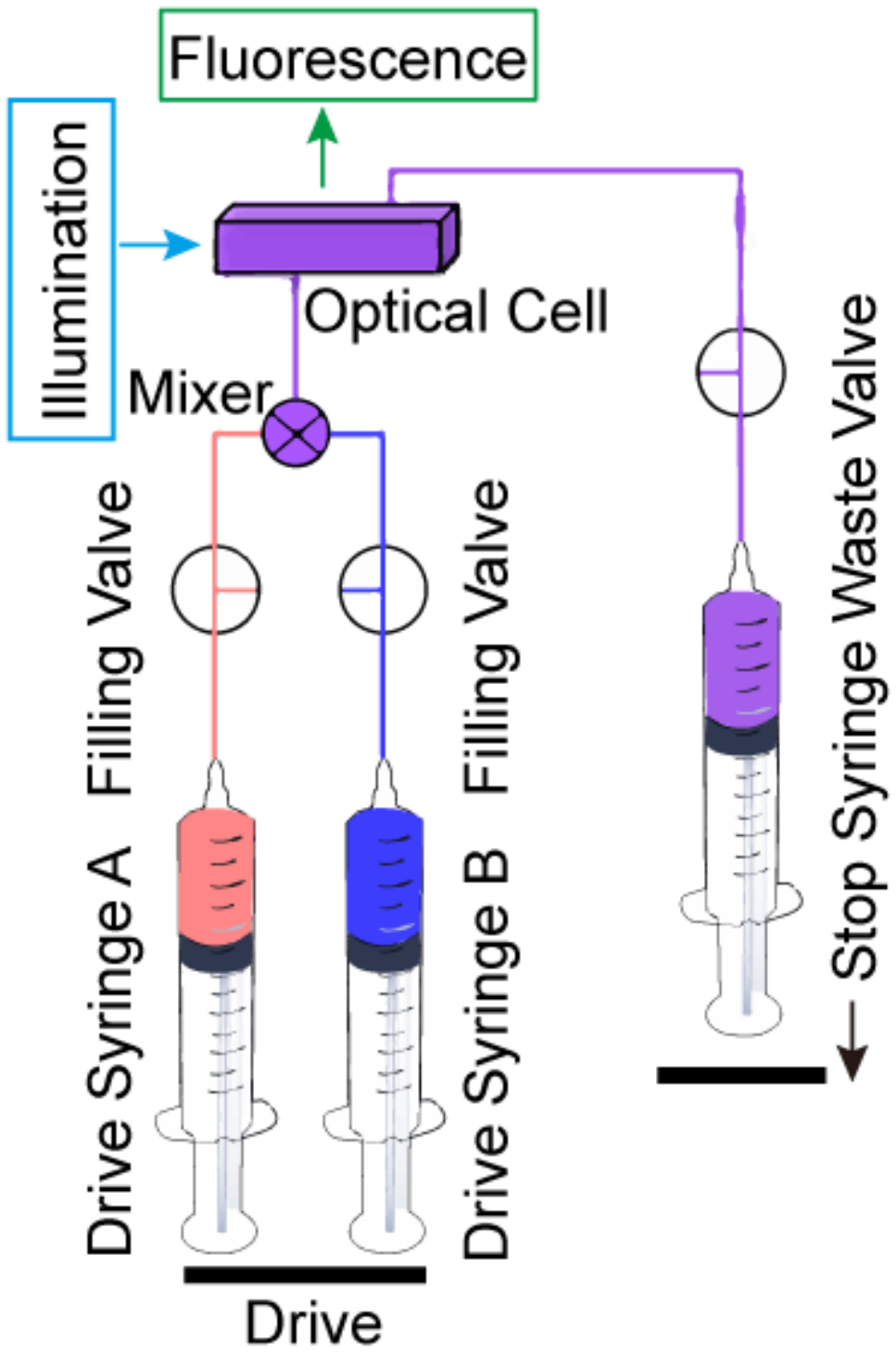

Figure S29. Schematic of the stopped-flow-based kinetics measurement.

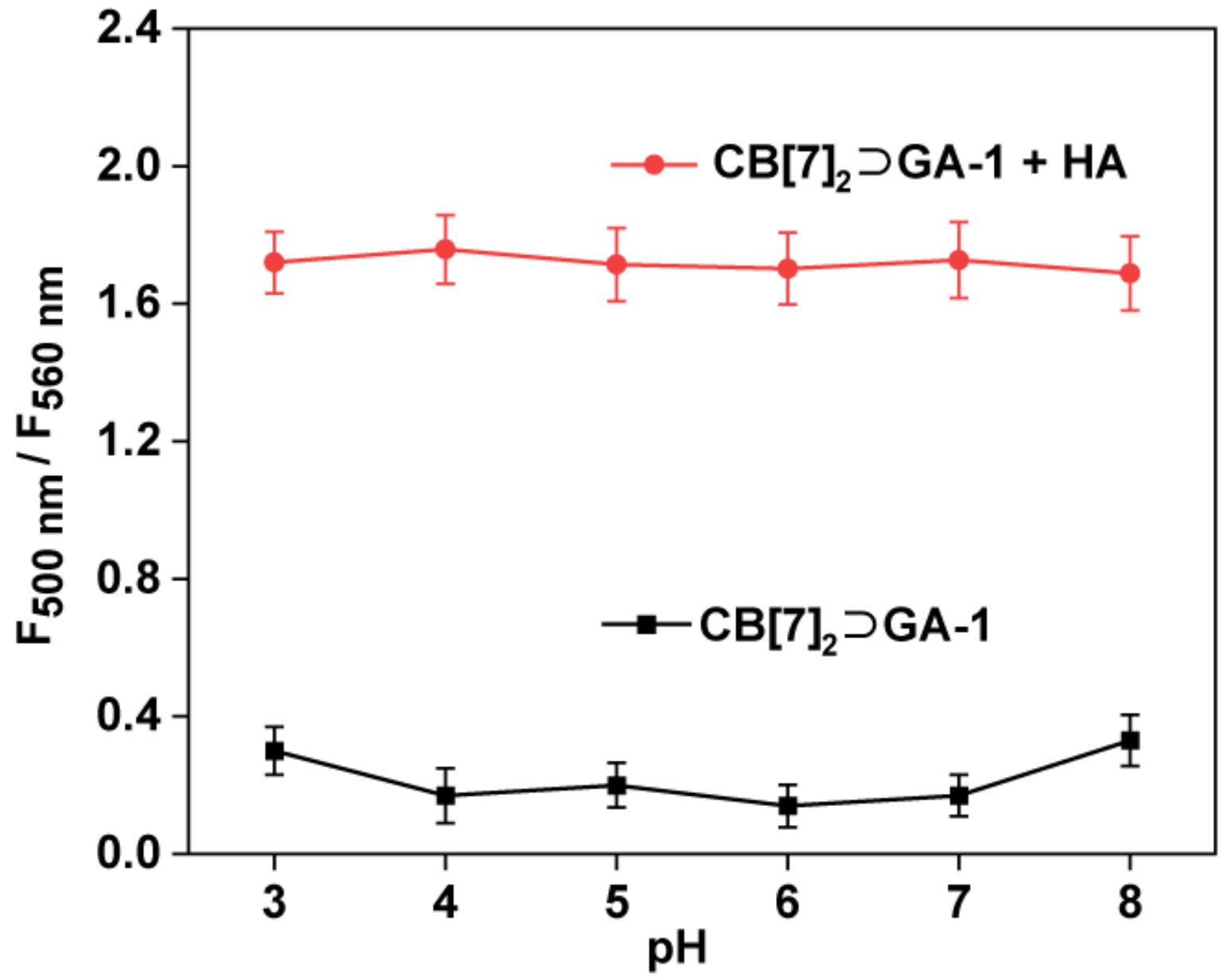

Figure S30. Fluorescence intensity ratios of **CB[7]$_2$⊃GA-1** (30 μM) at various pH values before (black) and after (red) reaction with HA (60 μM) at room temperature, excited at 440 nm.

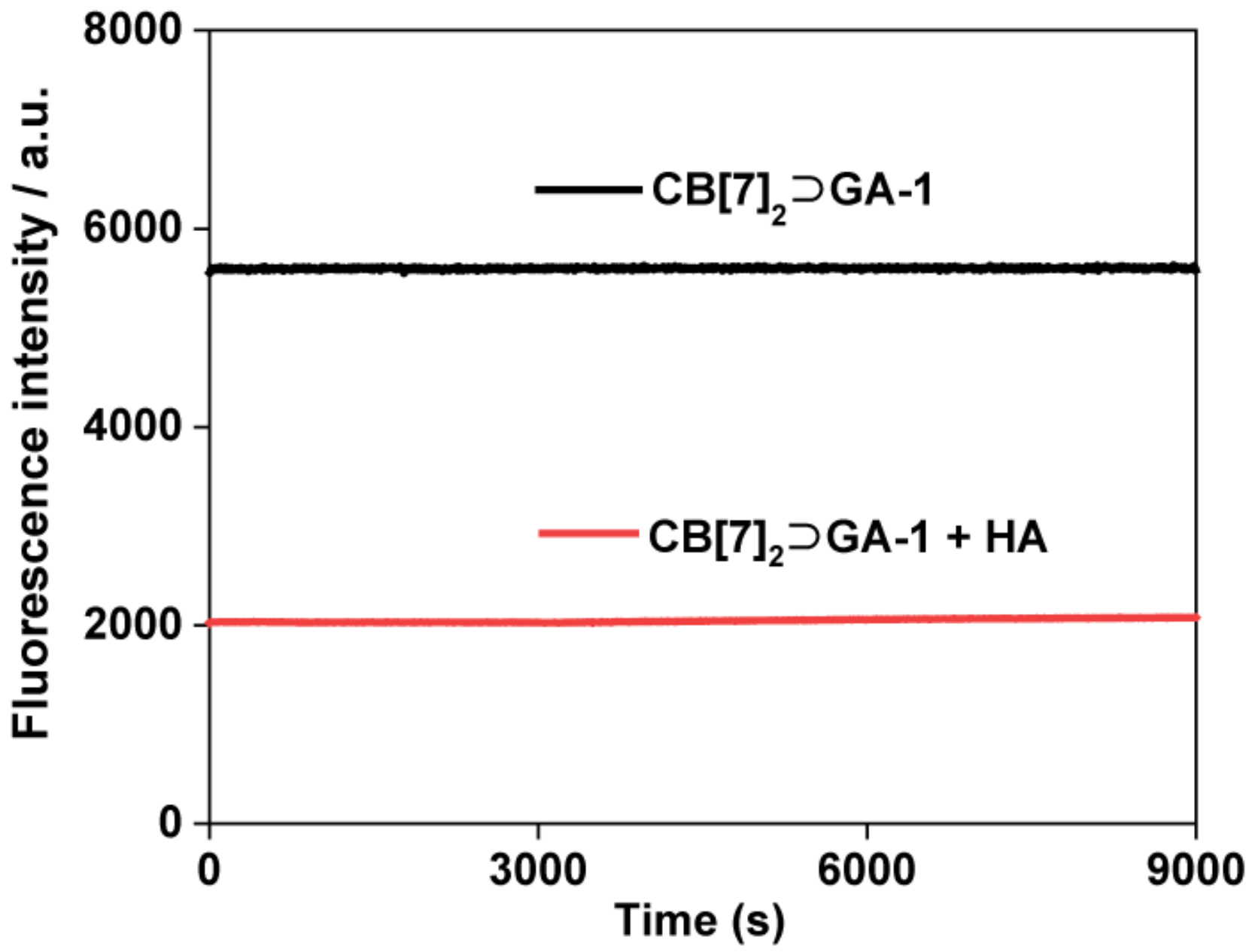

Figure S31. Photostability test of the **CB[7]$_2$⊃GA-1** and the product of **CB[7]$_2$⊃GA-1** reacted with HA, excited at 440 nm.

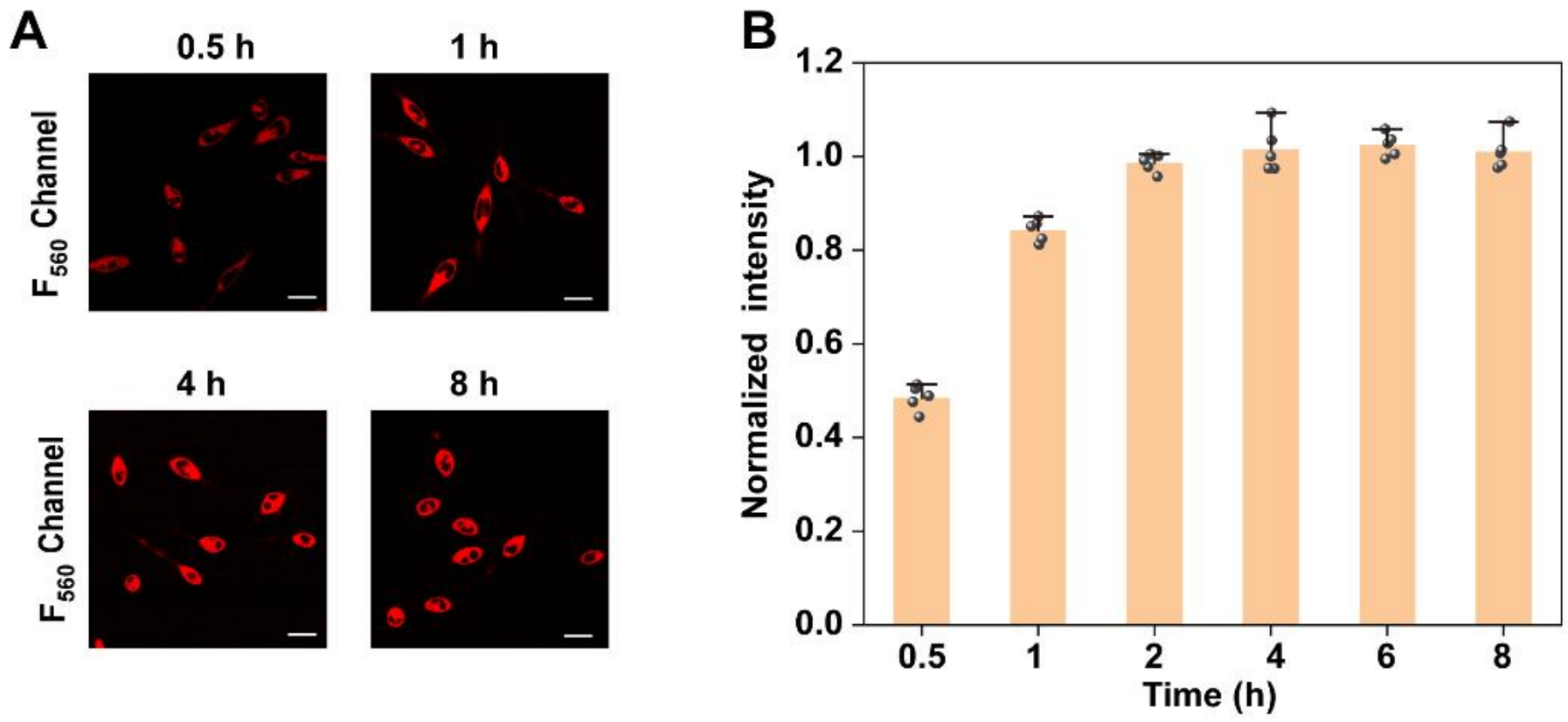

Figure S32. Cellular uptake kinetics of **CB[7]$_2$⊃GA-1** (30 μM) monitored by fluorescence spectroscopy. (A) Representative fluorescence images at selected time points (0.5, 1, 4, and 8 h). (B) Quantified normalized intensity versus incubation time (0.5-8 h). Data are presented as mean ± SD (n = 5), scale bar: 20 μm.

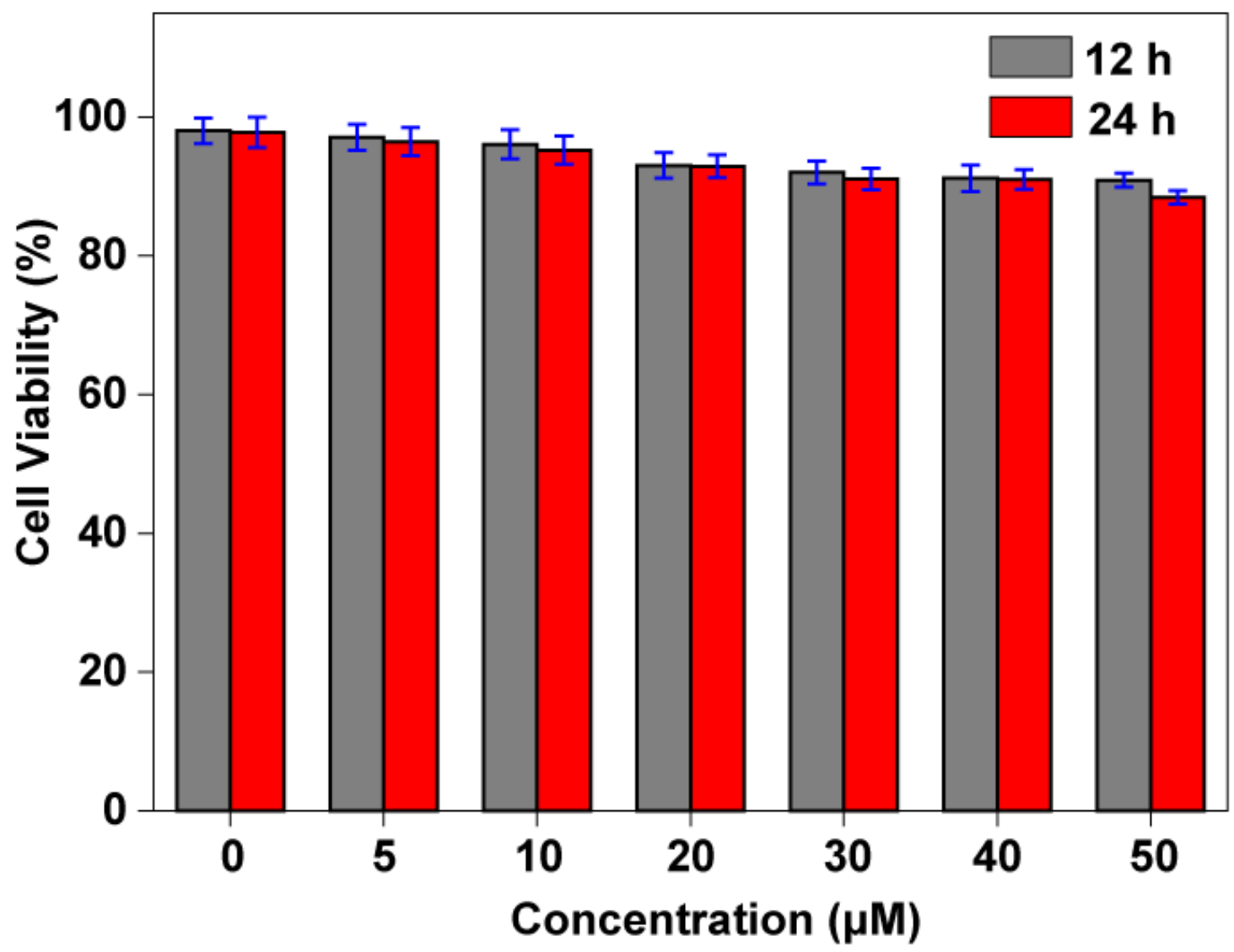

Figure S33. The MTT assay for mast cells upon incubation with **CB[7]$_2$⊃GA-1** at different concentrations (0, 5, 10, 20, 30, 40 and 50 μM) after 12 h and 24 h, respectively.

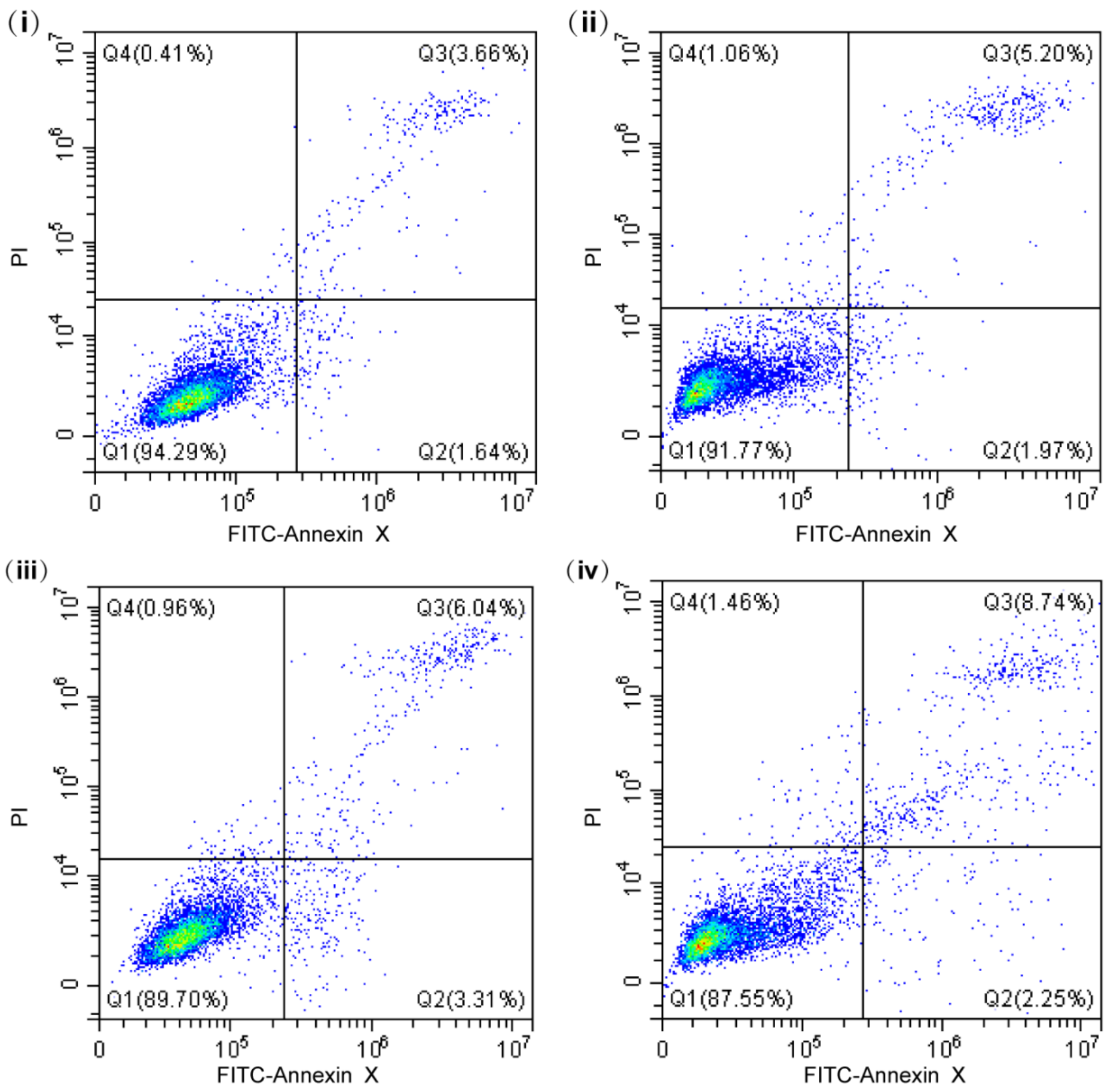

Figure S34. Apoptosis assay of mast cells after incubation with **CB[7]$_2$⊃GA-1** at concentrations of 0 (i), 10 (ii), 20 (iii), 30 (iv) μM for 24 h. Q1, Q2, Q3 and Q4 represent the regions of live cells, early apoptotic cells, late apoptotic cells and dead cells respectively.

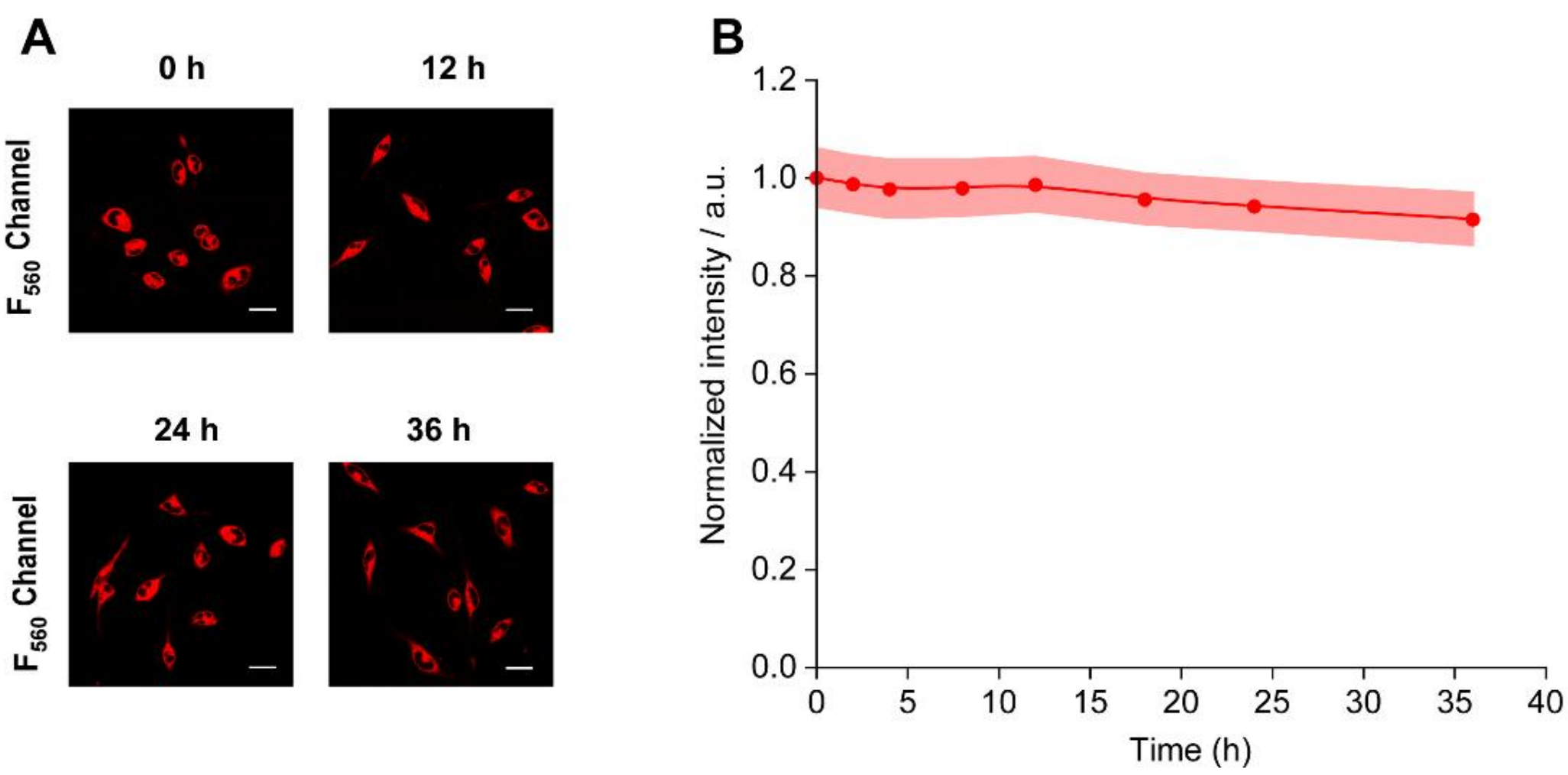

Figure S35. Confocal fluorescence imaging (A) and photobleaching profile (B) of **CB[7]$_2$⊃GA-1** in live mast cells, under 440 nm laser excitation. Scale bar: 20 μm.

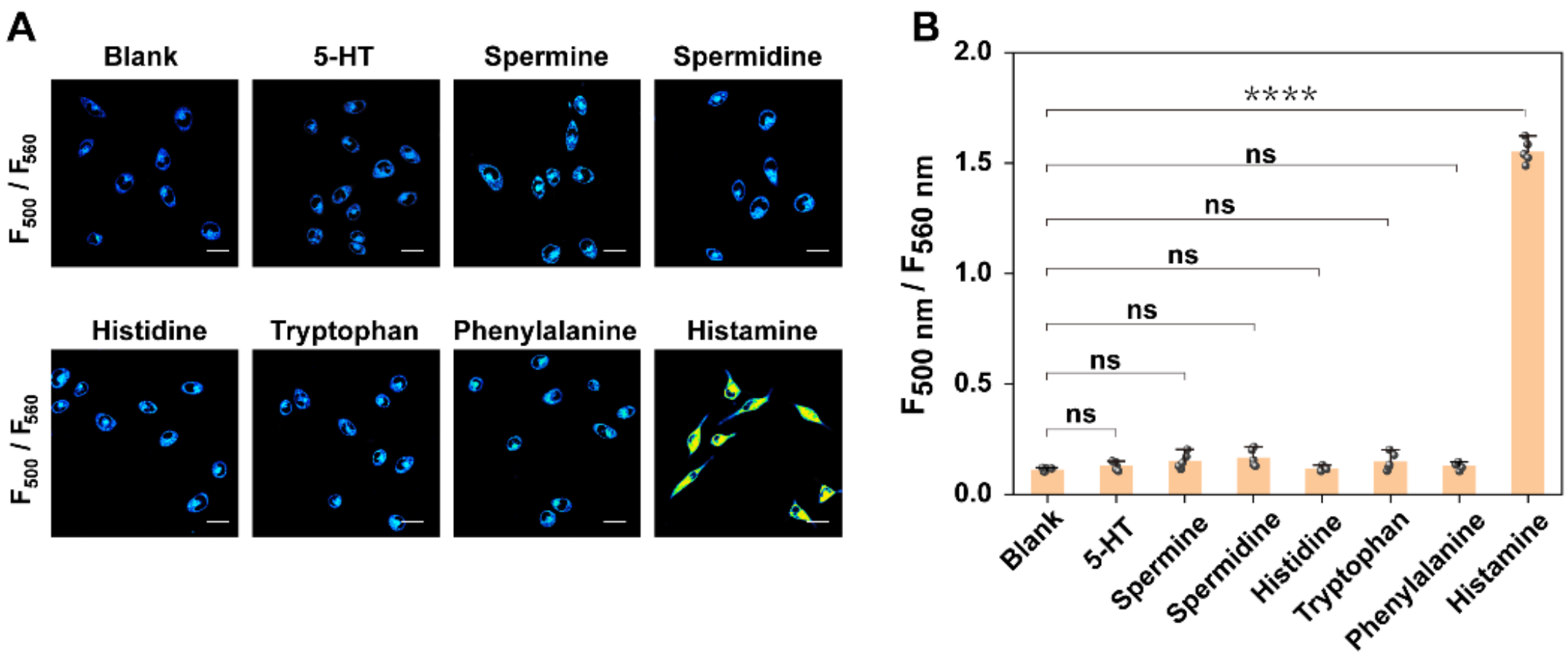

Figure S36. (A) Specificity assay of probe **CB[7]$_2$⊃GA-1** in mast cells. Cells were pretreated with 30 μM **CB[7]$_2$⊃GA-1** and subsequently incubated with different analytes. (B) Quantitative analysis of the mean fluorescence intensity corresponding to (A). Statistical significance (*****P*<0.0001) was determined by one-way analysis of variance (ANOVA). Data are presented as mean ± SD (n = 5), scale bar: 20 μm.

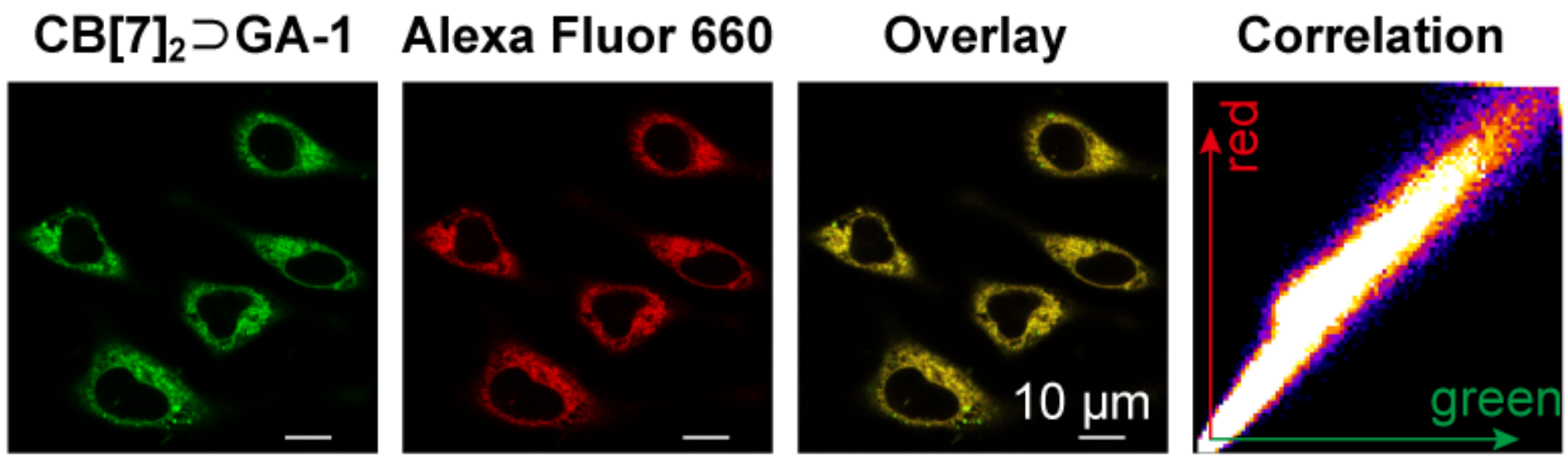

Figure S37. Co-localization imaging of probe **CB[7]$_2$⊃GA-1** with a commercial cytoplasm (Alexa Fluor 660); Pearson's correlation coefficients, ρ = 0.93. Scale bar: 10 μm.

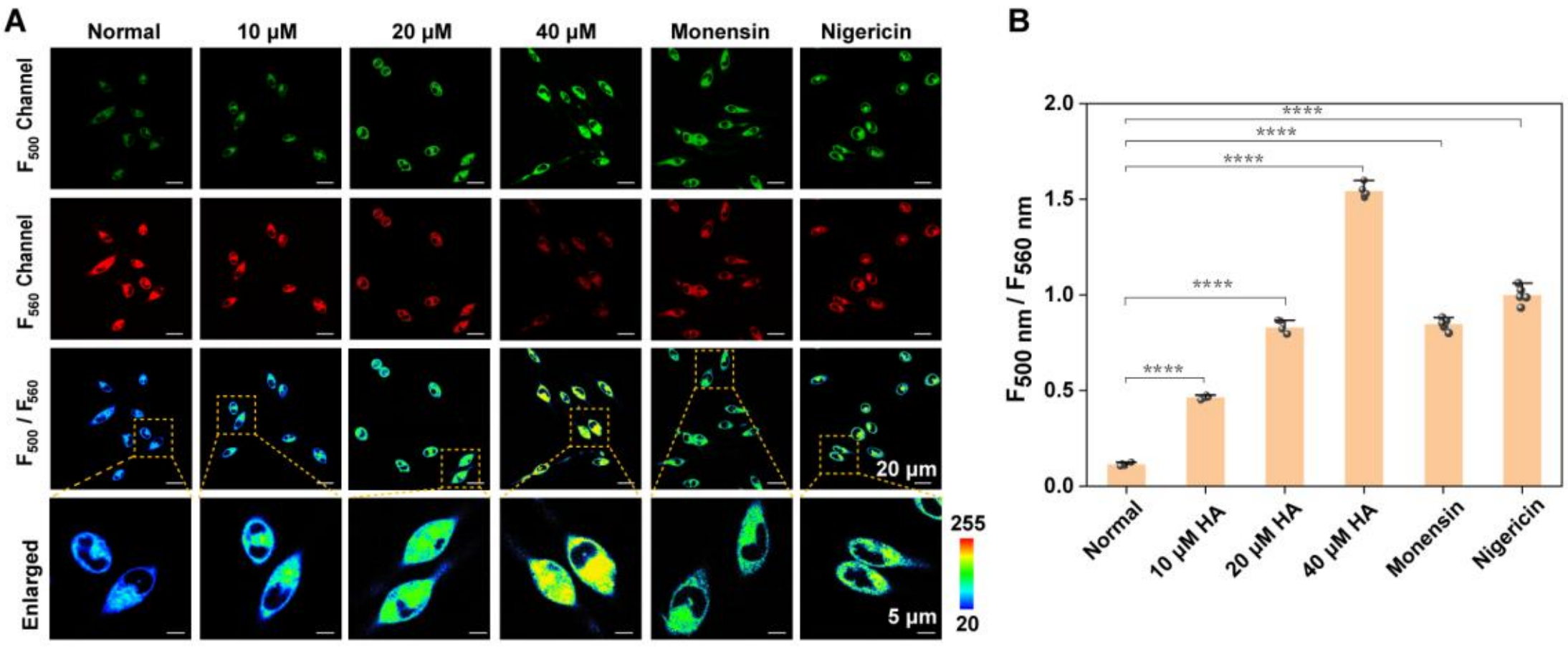

Figure S38. (A) Confocal fluorescence images of mast cells. Cells were preincubated with **CB[7]$_2$⊃GA-1**, and subsequently treated with different concentrations of HA (0, 10, 20, and 40 μM), or stimulated with 10 μM monensin and nigericin, respectively. [**CB[7]$_2$⊃GA-1**] = 30 μM, λ_{ex} = 440 nm. (B) Histogram of the relative fluorescence ratios ($F_{500\ nm}/F_{560\ nm}$) corresponding to panel (A). Statistical significance (*****P* < 0.0001) was determined by one-way analysis of variance (ANOVA). Scale bars: 20 μm (global images) and 5 μm (enlarged images).

4. References

(1) G. Kresse, J. Hafner, "Ab initio molecular dynamics of liquid metals," *Physical Review B* 47 (1993): 558-561.

(2) G. Kresse, J. Hafner, "Ab initio molecular-dynamics simulation of the liquid-metal-amorphous-semiconductor transition in germanium," *Physical Review B* 49 (1994): 14251-14269.

(3) J. P. Perdew, K. Burke, M. Ernzerhof, "Generalized gradient approximation made simple," *Physical Review Letters* 77 (1996): 3865-3868.

(4) G. Kresse, D. Joubert, "From ultrasoft pseudopotentials to the projector augmented-wave method," *Physical Review B 59* (1999)**:** 1758-1775.

(5) P. E. Blöchl, "Projector augmented-wave method," *Physical Review B* 50 (1994): 17953-17979.

(6) Becke A. D., "A Density-functional thermochemistry. III. The role of exact

exchange," *Journal of Chemical Physics* 98 (1993): 5648-5652.

(7) Weigend, F.; Ahlrichs, R, "Balanced basis sets of split valence, triple zeta valence and quadruple zeta valence quality for H to Rn: Design and assessment of accuracy," *Physical Chemistry Chemical Physics* 7 (2005): 3297-3305.

(8) Wadt, W. R.; Hay, P. J., "Ab initio effective core potentials for molecular calculations. Potentials for potassium to gold including the outermost core orbitals," *Journal of Chemical Physics* 82 (1985): 299-310.
